# Texture features in the Shearlet domain for histopathological image classification

**DOI:** 10.1186/s12911-020-01327-3

**Published:** 2020-12-15

**Authors:** Sadiq Alinsaif, Jochen Lang

**Affiliations:** EECS, University of Ottawa, Ottawa, Canada

**Keywords:** Texture descriptors, Histology, Complex shearlet, Classification, SVM

## Abstract

**Background:**

A various number of imaging modalities are available (e.g., magnetic resonance, x-ray, ultrasound, and biopsy) where each modality can reveal different structural aspects of tissues. However, the analysis of histological slide images that are captured using a biopsy is considered the gold standard to determine whether cancer exists. Furthermore, it can reveal the stage of cancer. Therefore, supervised machine learning can be used to classify histopathological tissues. Several computational techniques have been proposed to study histopathological images with varying levels of success. Often handcrafted techniques based on texture analysis are proposed to classify histopathological tissues which can be used with supervised machine learning.

**Methods:**

In this paper, we construct a novel feature space to automate the classification of tissues in histology images. Our feature representation is to integrate various features sets into a new texture feature representation. All of our descriptors are computed in the complex Shearlet domain. With complex coefficients, we investigate not only the use of magnitude coefficients, but also study the effectiveness of incorporating the relative phase (RP) coefficients to create the input feature vector. In our study, four texture-based descriptors are extracted from the Shearlet coefficients: co-occurrence texture features, Local Binary Patterns, Local Oriented Statistic Information Booster, and segmentation-based Fractal Texture Analysis. Each set of these attributes captures significant local and global statistics. Therefore, we study them individually, but additionally integrate them to boost the accuracy of classifying the histopathology tissues while being fed to classical classifiers. To tackle the problem of high-dimensionality, our proposed feature space is reduced using principal component analysis. In our study, we use two classifiers to indicate the success of our proposed feature representation: Support Vector Machine (SVM) and Decision Tree Bagger (DTB).

**Results:**

Our feature representation delivered high performance when used on four public datasets. As such, the best achieved accuracy: multi-class Kather (i.e., 92.56%), BreakHis (i.e., 91.73%), Epistroma (i.e., 98.04%), Warwick-QU (i.e., 96.29%).

**Conclusions:**

Our proposed method in the Shearlet domain for the classification of histopathological images proved to be effective when it was investigated on four different datasets that exhibit different levels of complexity.

## Background

In medical imaging, the study of histology images is considered a significant task [1]. The advancement of technology allows the histological slides to be digitized and stored in digital form [2]. The inspection of histological slides manually by a histopathologist is indispensable. However, computational techniques from image processing and machine learning can be of a great asset in the field of histopathology to assist in applying pre-screening/classification of easy cases. Therefore, more time can be consumed in studying the challenging histological slides. More importantly, computer-assisted diagnosis in histopathology can play a significant role in minimizing (and ultimately eradicating) man-made mistakes, e.g. by the pathologist [3].

Therefore, the early identification of cancer is crucial for the pathologist to propose an appropriate treatment for the patients. The process of histopathological tissue classification is tackled in different ways. We divide our review of such techniques into three groups: texture-based, Shearlet-based, and deep feature-based methods.

*Texture-based techniques* are frequently investigated for the analysis and classification of histopathological tissues. For instance, Kather et al. [4] proposed computing various texture features and classify colorectal cancer histology using SVM (i.e., using 10-fold cross-validation) into eight classes. The fusion of different texture features delivered an accuracy of 87.4%.

Similarly, Linder et al. [5] investigated a different number of descriptors: LBP, Haralick texture attributes, and Gabor filters to classify their introduced dataset which is called, Epistroma. As such, those extracted descriptors are fed to an SVM model to distinguish between epithelium and stroma tissues. Comparably, Spanhol et al. [3] established a new dataset called, breast cancer histopathology dataset (BreakHis). This dataset consists of benign and malignant tissues. Spanhol et al. used different techniques to classify BreakHis tissues into benign or malignant.

Bruno et al. [6] proposed applying LBP on the curvelet coefficients of the transformed image)(i.e., authors used the Digital Database for Screening Mammography, Breast Cancer Digital Repository, and UCSB biosegmentation benchmark). To reduce the number of descriptors, the authors used statistical analysis of variance (ANOVA). In comparison to Bruno et al., we also use descriptors computed from a directional wavelet transform, but we demonstrate that it is advantageous to integrate various descriptors computed in the Shearlet domain to create the feature space. A more relative idea to our technique is proposed by Ribeiro et al. [7]. Ribeiro et al. computed descriptors from both spatial images and curvelet coefficients to classify colorectal histology tissues. In contrast to Ribeiro et al., we utilized both magnitude and phase coefficients of the complex Shearlet domain. A similar approach to ours is proposed by Vo et al. [8] who extracted both, phase and magnitude descriptors for textured image retrieval, but applied to non-medical texture image samples.

*The Shearlet transform* has been previously used in different studies. Such a transform has the advantage of constructing an anisotropic system of a wavelet. However, only the magnitude coefficients are utilized for the classification of textured images. For instance, He et al. [9] classified textured images by proposing Shearlet-based descriptors. Authors in this study, quantize and encode the local energy descriptors computed from the Shearlet coefficients. Thereafter, the energy histograms of all levels are cumulated to form the image characteristics. Instead, Zhou et al. [10] utilized only specific levels of the decomposition of the Shearlet domain for breast tumor ultrasound image classification.

Dong et al. [11] suggested a technique for textured images classification and retrieval, where the dependencies of adjacent Shearlet subbands are modeled using linear regression. To represent the Shearlet subbands for classification, energy descriptors are computed. However, the textured image retrieval consists of both statistics in the contourlet and Shearlet domains.

Meshkini and Ghassemian [12] proposed to classify textured images using the inner product of the co-occurrence matrix and magnitude coefficients of Shearlet transform. Different to many published studies, we do not only use the magnitude coefficients, but also the phase coefficients of the Shearlet transform in our work.

*Deep feature descriptors* which are extracted from a pre-trained deep learning model (particularly, convolutional neural network (CNN)) which are typically trained on non-medical images. As such, these models are either used without fine-tuning (i.e., as unsupervised feature extractors) or fully/partially retrained for biomedical images. For example, Song et al. [13] proposed classifying the BreakHis dataset using feature vectors extracted from a CNN. As such, the extracted descriptors from the CNN are encoded using the Fisher Vector method. Similarly, Gupta et al. [14] extracted deep features from a fine-tuned DenseNet, but from multiple layers to classify BreakHis dataset. Differently, Wang et al. [15] utilized color deconvolution to obtain the hematoxylin and eosin channels separately. Subsequently, one CNN model is trained using hematoxylin components and another CNN trained using eosin components, and finally, the outputs of the two CNNs are fused to obtain the final prediction.

**Fig. 1:**
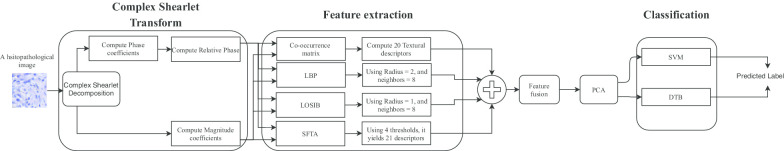
Summary of our work approach

As has been discussed above, computational techniques have been applied previously to predict the class of a tissue type in histological images. As such, conventional and deep learning (DL) techniques have been developed [16]. However, with the scarcity of well-curated histopathological datasets for training/testing deep neural networks  [17, 18], training a deep learning model can be a challenging approach. Therefore, in our study, we present a novel Shearlet-based hand-engineered texture descriptors to classify tissue types. Namely, co-occurrence texture descriptors [19, 20], Local Binary Patterns (LBP) [21], Local Oriented Statistics Information Booster (LOSIB) [22], and Segmentation-based Fractal Texture Analysis (SFTA) [23] are used in our study which each of these set of descriptors are computed in the Shearlet domain [24]. Then, these features are utilized to train/test two classifiers: Support Vector Machine (SVM) [25] and a Decision Tree Bagger (DTB) [26].

Notably, our modeling technique is taking advantage of the directionality in the complex Shearlet transform where we utilized both the magnitude and phase coefficients. As such, those coefficients are summarized using various textural methods that can capture local and global attributes [19, 23] of histopathological tissues. Most interestingly, computing such statistics from the directional sub-bands can potentially lead to capturing significant information that can be missed in the spatial domain because of the complexity of histopathological tissues.

In our research, we investigate both parametric and non-parametric (i.e., robust in classification [27]) classification models. We include DTB because it is a ML method that is considered to lead to explainable decisions, unlike SVM which is considered as a block box classifier [28]. We show that the fusion of some sets of descriptors can result in a vigorous feature representation. Thereafter, we employ principal component analysis to further enhance the classification results while having a reduced set of features.

**Fig. 2:**
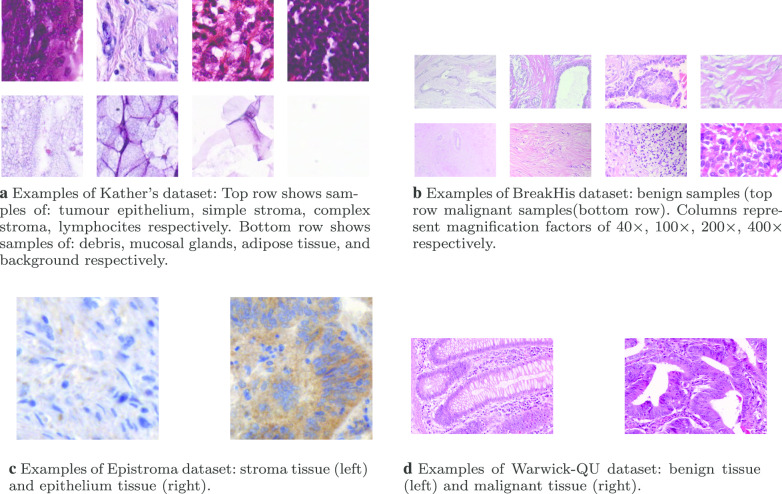
Samples of each dataset that we have used in our study

This paper is an extension of work previously presented at the Biomedical and Health Informatics (BHI) Workshop 2019 [29]. Our main contributions in this extension are summarized below:

- We propose and present a comprehensive justification for our feature space, namely, the Shearlet-based texture descriptors for histopathological image classification.
- We demonstrate that when these attributes are used to train a conventional ML model (i.e., SVM and DTB in this extended version), they provide better classification performance than several existing methods on the four standard datasets used in this research.
- We present an extended study of our feature representations expressed in principal components that decreases computational cost without significantly compromising accuracy.

## Methods

Figure 1 provides an overview of our proposed system for the classification of histopathological images. A detailed description of each component of our method is provided in the following sections. $\hbox {MATLAB}^{\textregistered }$ 2017b is utilized for the implementation of our techniques.

Our proposed method consists of three steps:

- Step 1: For a given histopathological image, we apply the complex Shearlet transform. With the complex coefficients, we calculate the magnitude and relative phase (RP).
- Step 2: We then extract four sets of features from the directional sub-bands of the RP and magnitude: co-occurrence based texture features, local binary pattern, local oriented statistics information, and segmentation-based fractal texture analysis.
- Step 3: We then apply one of two different classifiers: DTB or SVM.

### Datasets

Our proposed technique has been evaluated on four different histopathological datasets that exhibit different levels of complexity:

1. *Multi-class Kather’s* dataset [4] consists of a total of 5000 images (i.e., each image has a size of $150 \times 150$ pixels). This dataset provides tissue types that belong to 8-types (i.e., tumor epithelium, simple stroma, complex stroma, immune cells, debris, normal mucosal glands, adipose tissue, and background (no tissue)) - there are 625 tissue samples for each type. An example of each tissue type is shown in Fig. 2a.
2. *Breast Cancer Histopathological dataset (BreakHis)* dataset [3] contains tissue from two categories: benign and malignant breast tumors (Examples are shown in Fig. 2b). The total number of samples from two tissue types is 7909 images (i.e., each image has a size of $700 \times 460$ pixels). It worth noting that each histological slide is captured and stored with various magnification factors: $40\times$, $100\times$, $200\times$, and $400\times$. The distribution of samples of the benign/malignant in each magnification factor as follows: $625/1370 (40\times )$, $644/1437 (100\times )$, $623/1390 (200\times )$ and $588/1232 (400\times )$. Spanhol et al. [3] proposed to use each magnification factor as a separate dataset. However, in our study, we combine all magnification factors as one dataset as Jonnalagedda [30]. This is motivated by the fact that each magnification factor captures different information [31].
3. *Epistroma* dataset contains variable size histo-pathology images that belong to two tissue types (as shown in Fig. 2c): stroma (551 samples) and epithelium (825 samples) [5].
4. *Warwick-QU* dataset obtained with a magnification factor of $20 \times$ from colon histology sections. It is a binary dataset of benign (74 samples) or malignant (91 samples) [32]. Examples of the tissue types are shown in Fig. 2d.

### Complex Shearlet transform

In this study, we present a new perspective for computing attributes that summarize the statistical information/distribution of the Shearlet magnitude/phase coefficients for every scale and orientation [24]. Given a complex coefficient, $C = x + iy$ where first term express the real part and the second term express the imaginary part, then we can compute the magnitude (as $\rho = \root 2 \of {x^2 + y^2}$) and phase (as $\theta = tan^{-1}(y/x)$) components. In contrast to existing studies [9, 10], we do not only use the energy of the complex Shearlet transform, but we also examine the usefulness of the phase components and their potential for providing more robust characterization for medical image classification. We experimentally verify that using phase alongside with magnitude coefficients can, in fact, boost the classification performance (See Section ).

Our work is motivated by research completed by Vo et al. [8]. Vo et al. build a feature space (i.e., consist of magnitude and relative phase (RP)) that is computed from a complex directional filter bank for textured image retrieval. However, In our study, we acquire such an idea but alternatively compute the relative Shearlet phase components. The complex Shearlet transform can be applied on a histopathological image that has a size of $M \times M$ to be transformed to *S* scales where every scale consists of *K* directionalities. Let $\theta _{sk}(i, j)$ at location (*i*, *j*) to represent the phase angle component at at scale *s* and directionality *k*, where $s = 1, 2, ..., S$ and $k = 1, 2, ..., K$. In our study, we use $S = 4$ and $K = 8$ per scale.

Now, we can compute the relative Shearlet phase for a phase component at position (*i*, *j*) of a directional sub-band in the following manner:

1 $$\begin{aligned} RP_{sk}(i, j) = {\left\{ \begin{array}{ll} \theta _{sk}(i, j) - \theta _{sk}(i, j + 1), &{} {\text {if } 1 \leqslant k \leqslant \frac{K}{2}}.\\ \theta _{sk}(i, j) - \theta _{sk}(i + 1, j), &{} { \text {if } \frac{K}{2} < k \leqslant K}. \end{array}\right. } \end{aligned}$$

We choose the differences of vertical and horizontal because of the orientation of shearing in the Shearlet transform. Such a transform has the advantage of being multi-scale and multi-directional which in turn can be a significant tool for a multi-resolution analysis of histopathological tissues. Therefore, we utilize each directional sub-band (i.e., from both magnitude and RP) to calculate statistical attributes.

The Shearlet coefficients localize spatially distributed discontinuities and are contrast invariant [33]. Although curvelets [34] and contourlets [35] have similar properties to Shearlets and have been used for image classification [6, 7]. However, both have certain limitations [24].

In this study, we use a publicly available implementation of complex Shearlet transform, called the ShearLab [36]. After each histology image is transformed, we then summarize the magnitude and RP of the Shearlet components using four various techniques. Each technique is briefly detailed as follows:

#### Co-occurrence matrix (CM)

The CM was introduced by Haralick et al. [20]. Later, other studies presented other types of statistics that can be computed from the CM [19, 37]. However, given two pixels (i.e., *i* and *j*) that are apart from each other by a distance (*PD*), then the content of a gray-level image can be formulated as a relative frequencies (i.e., $F_{ij}$) matrix. Also, there is another hyper-parameter that can be adjusted while computing the CM which is at which orientation to compute the relative frequencies. As a result, we have a CM that consists of relative frequencies for quantized orientation and distance between neighboring pixels.

In our application of CM on the directional sub-bands of Shearlet coefficients, we calculate the CMs using a constant distance of $PD = 1$, but changing the orientation = ($0^{\circ }$, $45^{\circ }$, $90^{\circ }$, and $135^{\circ }$); hence, we have four CMs. To obtain rotation invariant statistics from the CMs, we compute the mean of those four CMs [4]. Thereafter, from this CM, we extract twenty textural features (i.e., contrast, correlation, energy, autocorrelation, cluster prominence, cluster shade, dissimilarity, entropy, homogeneity, maximum probability, sum of squares, variance, sum average, sum variance, sum entropy, difference variance, difference entropy, information measure of correlation, inverse difference normalized, and inverse difference moment normalized). Following such a common practice to compute those statistical information out of CM for each magnitude/RP components, we then have a total of 640 attributes for each magnitude and RP directional sub-bands (i.e., $20 \times 32 (\#\text { of directional subbands)}$.

In our study, we obtain the CM for the Shearlet magnitude and RP coefficients for each directional sub-band instead of the spatial domain. In the spatial domain, CM analyzes the statistical information of the gray-level image, but in our application, we analyze the crucial directionality characteristics in the Shearlet domain which potentially leading to robust feature space for histopathological image classification.

#### Local binary pattern (LBP)

The LBP [21] texture attributes are rotationally invariant of the local occurrence of the gray-level of an image. Such, occurrence is naturally classified as ‘uniform’ patterns. Again, we are rather concerned with the Shearlet’s magnitude and RP coefficients of a histopathological image (i.e., can exhibit complex patterns). Therefore, utilizing the Shearlet coefficient which encapsulates field dominant direction chraterstics [38, 39] can potentially lead to robust summarization of such directionalities (i.e., represent crucial structural details, e.g., step edges [40]).

Hence, we apply the LBP on the magnitude and RP of each directional sub-band of the Shearlet coefficients to encode the field dominant directions. For a given Shearlet (SH) coefficient (i.e., representing the magnitude or RP) at the location (*i*, *j*), the LBP of the SH coefficient is computed as follows:

2 $$\begin{aligned} LBP_{P,R} = \sum _{p=0}^{P - 1} s(SH_p - SH_c)2^{p}, s(x) = {\left\{ \begin{array}{ll} 1, &{} {x \geqslant 0}.\\ 0, &{} {x<0}. \end{array}\right. } \end{aligned}$$

$SH_c$ and $SH_p$ are the central RP (or magnitude) values, and *P* represents the surrounding RP (or magnitude) values in the circular neighborhood.

In our application of LBP, we compute a feature vector for every directional sub-band while utilizing a radius of $R = 2$ and a neighborhood of $P = 8$, where the window size is set to be equal to the directional sub-band size. Therefore, we obtain a feature vector for each sub-band of length of $(P + 2)$ [21]. After concatenating all feature vectors of all directional sub-bands, we get a feature vector representing a histopathological image of length $10 \times 32 = 320$ attributes for each magnitude and RP.

#### Local oriented statistic information booster (LOSIB)

The LOSIB [22] technique first compute the absolute difference $d_p$ for a given central Shearlet coefficient with *P* neighboring coefficients (i.e., completed for all central magnitude (or RP) coefficients *c* of a directional sub-band) as follows: $d_p(i_c, j_c) = |SH_c - SH_p|$ where $p \in {0, 1, ..., (P - 1)}$.

Then, the mean of the differences across the same directionality is computed, as follows:

3 $$\begin{aligned} \mu _p = \frac{{\sum _{x_c = 1}^{M}}{\sum _{y_c = 1}^{N} d_p(x_c, y_c)}}{M \cdot N} \end{aligned}$$

where *N* and *M* represent the height and width of the image, respectively.

In our application of LOSIB, we set the radius $R = 1$ and neighborhood $P = 8$. Therefore, we obtain a feature vector for every directional sub-band of length *P*. However, the total number of descriptors for each of the magnitude and RP is $8 \times 32 (\#\text { of directional subbands)} = 256$.

#### Segmentation-based fractal texture analysis (SFTA)

Our application of SFTA on the directional sub-bands is adopted as provided by Costa et al. [23]. As such, SFTA first process the input directional utilizing a Two-Threshold Binary Decompositions (TTBD). As a result, various binary images are generated from the following attributes are computed: the dimension of the fractal boundaries, the average gray level, and the count of pixels belonging to the region.

In our utilization of the technique by Costa et al. [23], we set the number of thresholds to $n_t = 4$. As a result, for every directional sub-band, we get a 21 attribute. Hence, in total, we have $21 \times 32 (\#\text { of directional subbands)} = 672$ attributes for each, magnitude and RP.

### Fusion of feature sets

The aforementioned set of descriptors are examined individually for their robustness while being used for training a classifier. It is worth noting that each set of descriptors captures different intrinsic statistical information. Therefore, we examine some combinations of our descriptors which we expect to improve the classification performance. We investigate the following combinations:

- Fusion #1: (CM + LBP + LOSIB + SFTA) descriptors of Shearlet RP and/or magnitude.
- Fusion #2: (CM + LOSIB) descriptors of Shearlet RP and/or magnitude.
- Fusion #3: (CM + LBP + LOSIB) of Shearlet RP and (CM + LBP + SFTA + CM Dot Shearlet coefficients [12]) of Shearlet magnitude.

The aforementioned combinations are selected based on the merit of the individual set of features leading to good classification results.

### Principal component analysis (PCA) for feature reduction

In the previous section, we investigate feature fusion. Evidently, with the combination of different descriptors, the number of features increases. Therefore, the best achieving fusion strategy is processed with PCA to find a reduced subspace. The PCA coefficients are rotated to maximize the orthomax criterion [41], and to obtain a final basis with simple structure [42]. Being the principal components (PCs) rotated to maximize varimax, then they are utilized to project the combined descriptors to a decorrelated space.

### Classification algorithms

In this work, we introduce a new enhanced technique for analysis and feature extraction from the Shearlet transform. We also analyze the performance of two classifiers for different tissue types and show that the classifier has a minimal effect once robust features are extracted. In particular, we consider one of widely used in many of bio-medical research: a Support Vector Machine (SVM). In addition to SVM, we consider one type of decision tree which is a Decision Tree Bagger (DTB) [26] that has the trait of being non-parametric. As such, the distance between feature vectors is not computed in constructing decision trees. In contrast, SVM performance is based on kernels which can influence the classification performance [43]. Both SVM and DTB are briefly described below:

#### Support vector machine (SVM)

In our study, we utilize a SVM with pairwise classification (i.e., one-versus-one class decisions) [44]. Prior to training, we normalize all feature vectors to have equal mean and variance. The kernel function transforms the input data into a higher-dimensional feature representation, from which a hyperplane is formulated for classifying the input tissue dataset.

SVM training requires the solution of a quadratic programming (QP) problem. To simplify the solution of the problem, a sequential minimal optimization (SMO) solver is used in our study [45]. This solver simplifies the QP into a smaller series of QP for the training of SVM.

SVM training is impacted by the choice of the kernel function. In our study, we choose a radial basis function as a kernel function as it universally approximates the training dataset accurately. Therefore, the regularization parameter (C) is the only remaining parameter that can be optimized. In the attempt of choosing the best value of C, trials of values between [1, 5] with an increment of 1 were conducted. We found that in general $C = 5$ delivered the best classification performance on the testing dataset. Therefore, the value of $C = 5$ is set for all of our experiments. More details about SVM can be found in [46].

#### Decision tree bagger (DTB)

DTB is an ensemble classifier where each classifier in the ensemble is a classic decision tree generated using a random selection of attributes at each node to determine the split as in a random forest [47]. DTB generates multiple bootstraps (i.e., replicas of the training set) to train a decision tree with replacement from the provided training set. Thereafter, a bagging technique is used to combine the results of several decision trees, as an advantage this minimizes the susceptibility to over-fitting.

**Fig. 3:**
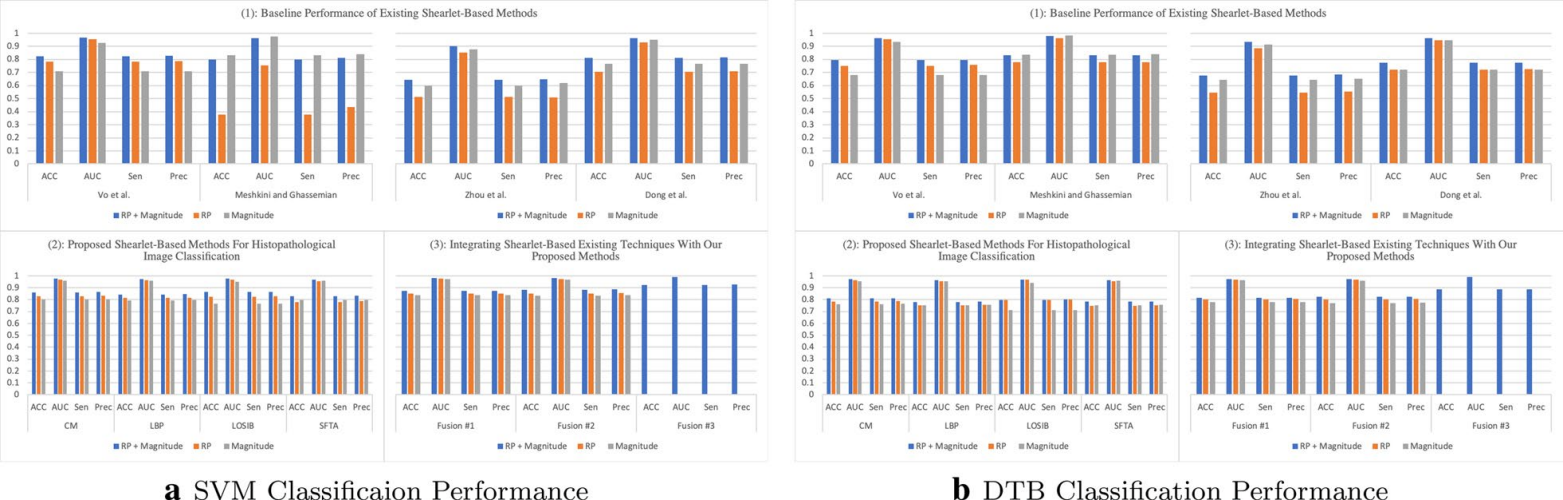
Classification performance on Kather’s dataset

**Fig. 4:**
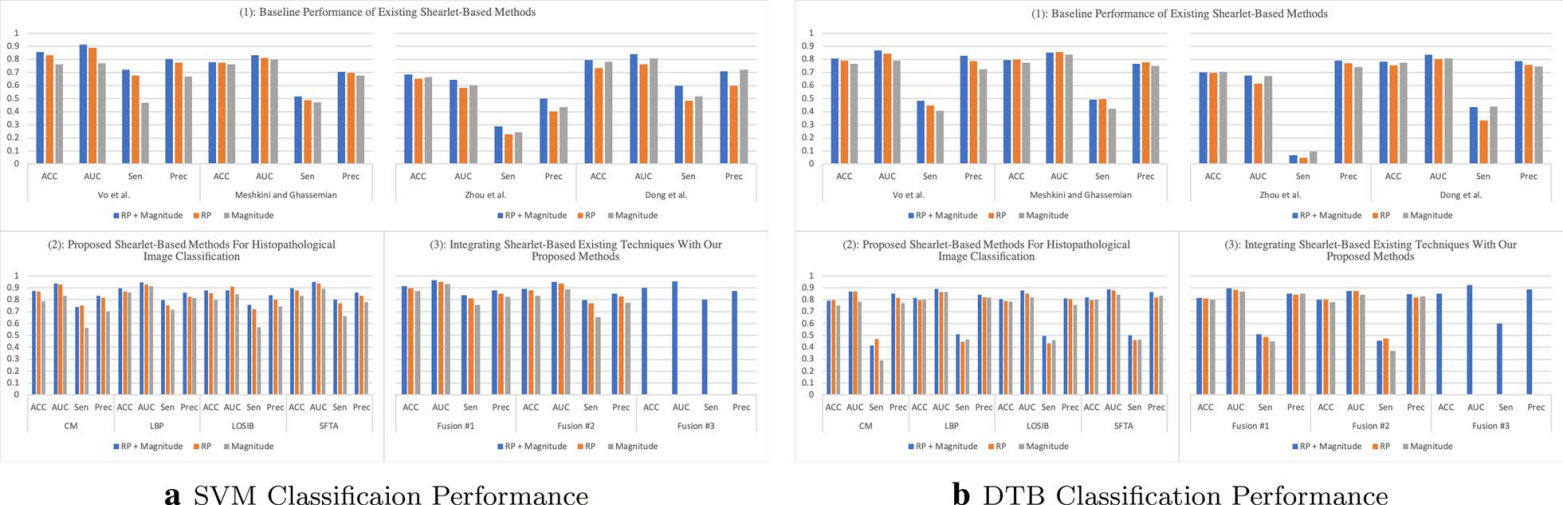
Classification performance on BreakHis dataset

### Cross-validation (CV)

We utilize cross-validation (CV) to obtain robust statistical results and to be able to generalize the classification results with each classification model. This approach is commonly used in the literature to examine a proposed technique. Therefore, our choice of the number of folds to split a dataset is based on previous studies. As such, the following dataset are divided using 10-fold CV: **multi-class Kather’s**(as Kather et al. [4]), **Epistroma**(as Ramalho et al. [48]), **Warwick-QU**(as Ribeiro et al. [7]).

The datasets are divided into mutually exclusive folds with approximately equal size (i.e., some of the datasets are imbalanced): 9-folds are used to build a model, and 1-fold is used to test the model. The process is repeated 10-times, such that the test set is different each time. Finally, the overall performance metrics are estimated by taking the mean from the tested 10 independently built models. However, the **BreakHis** dataset is split into 7-folds CV for training and testing as in a previous study [30].

**Fig. 5:**
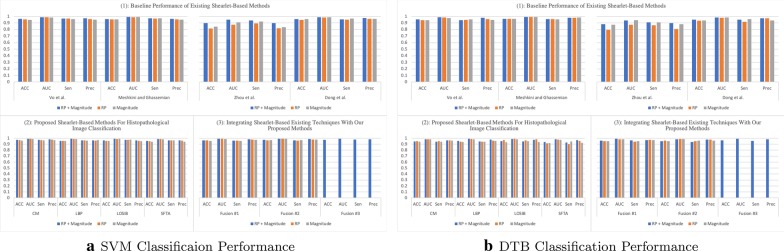
Classification performance on epistroma dataset

**Fig. 6:**
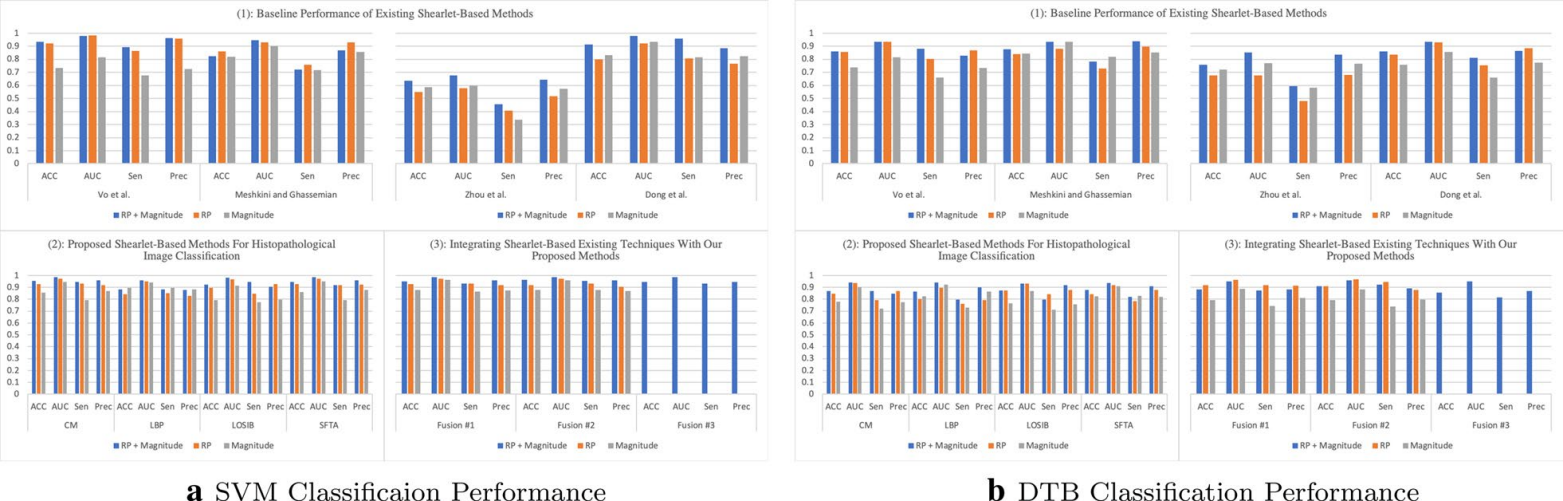
Classification performance on Warwick-QU dataset

#### Performance metric

To evaluate the performance of each built model, commonly used measures are computed in our study: accuracy (ACC), AUC (area under the receiver operating characteristic (ROC) curve), sensitivity (Sen), and Precision (Prec). The computation of each evaluation metric is calculated as follows:

- $ACC = TP + TN/N$. This metric represents the test examples that are correctly classified over all the number of test examples available. As such, true positive (TP) and true negative (TN) are the correct examples, and $N = (TP + TN) + (FP + FN)$, where false positive and false negative are denoted as FP and FN, respectively.
- AUC: This metric relies on the plot of ROC (i.e., false-positive rate (=FP/(TN+FP)) on the x-axis against sensitivity on the y-axis). Therefore, the accumulated area under the ROC curve yields the AUC value (i.e., between 0 & 1, where 1 is a good performing model).
- $Sen = TP/(TP+FN)$ measures the ability of a model to correctly predict the positive condition, when it is actually positive.
- $Prec = TP/(TP+FP)$ measures the ability of a model to correctly identify positive cases.

## Results and discussion

We first apply the Shearlet transform as detailed previously to four different datasets. We compute the RP and magnitude from the complex Shearlet coefficients. Then, to evaluate the strength of our proposed techniques, we utilize four commonly used measures for classification: ACC, AUC, Sen, and Prec.

In the literature, there exist descriptors extracted from multi-directional and multi-resolution wavelets (e.g., Shearlet, contourlet, and others). In this regard and to the best of our knowledge, we have implemented descriptors of previously published studies. These descriptors are applied on the transformed (i.e., using complex Shearlet transform) histopathological dataset:

- Vo et al. [8]: proposed to compute from the RP the circular mean and circular variance, but computed only the mean from the magnitude of the decomposed textured image while using a complex directional filter bank. However, we adopt the same descriptors, but we compute them from each of the Shearlet directional sub-band and then concatenate them to form the feature vector.
- Meshkini and Ghassemian [12]: proposed first to find the magnitude of the Shearlet coefficients and gray-level co-occurrence matrix. Then, the inner product of both is used as a feature vector.
- Zhou et al. [10]: proposed to compute three sets of descriptors from the magnitude coefficients only: (1) the co-occurrence matrix is computed (i.e., from which the following texture features are obtained: entropy, correlation, contrast and, energy) from the first layer only of the horizontal cone of Shearlet transform; (2) the mean, variance, and energy are calculated from the Shearlet transform, only, from the first and third layer in the horizontal and vertical cones; (3) The maximal values are obtained from each column only from the high-frequency of the Shearlet transform. Then, these three sets of descriptors are concatenated to form the feature vector.
- Dong et al. [11]: proposed to calculate from the Shearlet magnitude coefficients the following statistics: the mean and standard deviation. As such, these statistics are computed from each directional sub-band and concatenated to form the feature vector of an image.

Part (1) in Figs. 3, 4, 5, and 6 presents the classification performance of the techniques as detailed above for Kather, BreakHis, Epistroma, and Warwick-QU datasets, respectively. Further, we provide the classification results in form of tables in the appendix (i.e., the variants shown in italic in Part (1) of the Tables 1, 2, 3, and 4 are not examined in the original research papers but we have included them for comparison). We notice that utilizing both of the magnitude and RP of the Shearlet transform enhance the classification performance irrespective of the classifier (i.e., SVM or DTB) model and enhances the performance of other techniques. As such, the attributes proposed by Vo et al., using both magnitude and RP, attain exceeding performance than other baseline techniques.

When utilizing the multi-class Kather’s dataset, our proposed descriptors computed from the Shearlet coefficients of both magnitude and RP can accomplish reasonable accuracy between  82% to 86% when using an SVM model, but DTB yields accuracy spans only between  79% to 81% as presented in Part (2) of Fig. 3 (See Table 1). The highest AUC value = 0.9773 is obtained while classifying Kather’s dataset utilizing LOSIB attributes coupled with the SVM model. Furthermore, we compute the Sen and Prec which the highest values are achieved using LOSIB coupled with SVM (i.e., 0.8632 & 0.8664, respectively). However, when incorporating various descriptors together, for instance **Fusion #3** improves the accuracy about 6.22% points as presented in Part (3) of Fig. 3 & Table 1 when using an SVM model.

We outline the classification performance on the BreakHis dataset, such that we consider all four magnification factors as one dataset (as seen in Fig. 4). We observe that SFTA has attained the highest accuracy of 89.72% on the validation split (with a corresponding AUC = 0.9527, Sen = 0.8040, and Prec = 0.8593) while utilizing both magnitude and RP. As presented in Part (3) of Fig. 4 (See also Table 2), **Fusion #1** has led to a higher accuracy of 91.28% (with an AUC = 0.9650, Sen = 0.8391, and Prec = 0.8775). Evidently, due to the high skewness in the BreakHis dataset, the classifier is biased toward the majority class (i.e., malignant cases) as it is observed with lower values in sensitivity and precision.

Similarly, we conduct our technique on the Epistroma dataset (results reported in Fig. 5 & Table 3). We observe when descriptors extracted from both, magnitude and RP, utilizing CM leads to an accuracy of 97.24% with AUC = 0.9917, Sen = 0.9733, and Prec = 0.9807. In Part (3), **Fusion #3** enhances the accuracy to 97.46% with a AUC value = 0.9925, Sen = 0.9769, and Prec = 0.9809.

Moreover, the classification performance on Warwick-QU are is presented in Fig. 6 (See also Table 4). Once more, CM textural features of both, magnitude and RP lead to the best accuracy of 95.70% (with corresponding AUC = 0.9860, Sen = 0.9446, and Prec = 0.9589) of our proposed individual feature representation. Part (3) presents that **Fusion #2** leads to the highest accuracy of 96.29% with an AUC = 0.9860, Sen = 0.9571, and Prec = 0.9589. It is worth noting that the outlined standard deviations corresponding with classification performance on Warwick-QU dataset are higher than the other datasets because the number of validation examples of this dataset is significantly smaller.

**Fig. 7:**
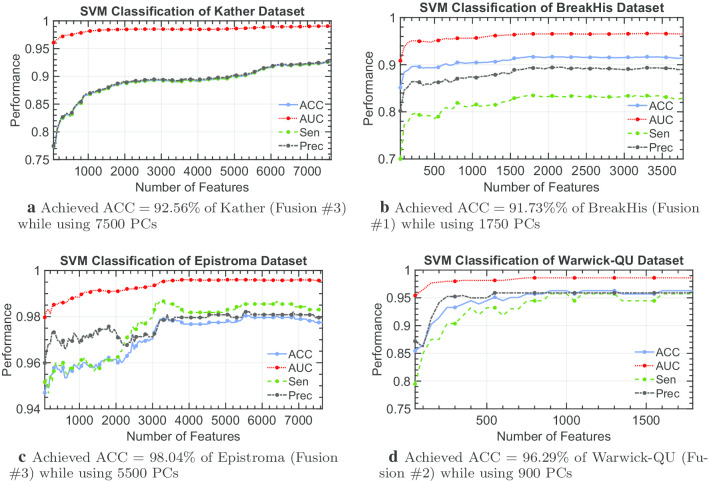
Classification performance of SVM with an increment of 50 principal components (PCs) of best fusion till all PCs are used

**Fig. 8:**
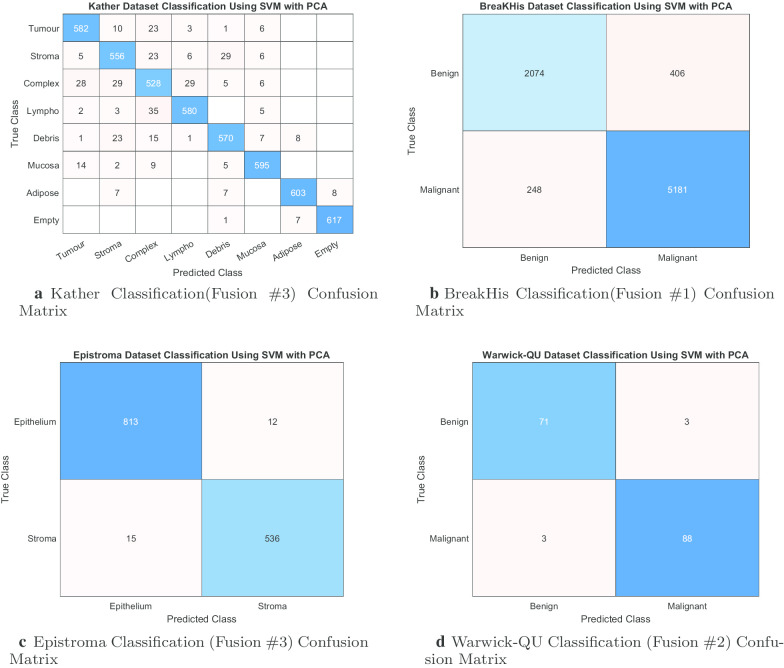
Confusion matrices of our best performing proposed methods while using reduced set of features (See Fig. 7)

Further, Fig. 7 shows the classification performance while attempting to find a reduced set of our feature space for each dataset. In the previous sections, we have identified the best achieving fusion descriptors. Therefore, we further process these descriptors with PCA. As such, we aim to retrieve the PCs that maintain or improve the classifier performance in comparison to using all attributes. We have established from Tables 1, 2, 3 and 4 that the SVM model has strong capabilities to classify histopathological tissues which therefore coupled with the best fusion of the corresponding dataset to find a reduced set of features. However, we are able to utilize a reduced set of descriptors to classify multi-class Kather’s dataset with an accuracy of $92.56\% (\pm 1.29\%)$ (with a corresponding AUC = $0.9905(\pm 0.0020)$, Sen = $0.9256 (\pm 0.0130)$, and Prec = $0.9270 (\pm 0.0125)$) while using only 7500 descriptors (out of 7648) from Fusion #3. For classifying BreakHis dataset, a drastic pruning of descriptors (i.e., only 1750 out of 3776 are needed) while somewhat enhancing the classification accuracy to = $91.73\% (\pm 00.59\%)$ with AUC = $0.9654 (\pm 0.0057)$, Sen = $0.8363 (\pm 0.0188)$, and Prec = $0.8936 (\pm 0.0169)$. Again, the classification accuracy of Epistroma improves rather to $98.04\% (\pm 1.03\%)$ with AUC = $0.9960 (\pm 0.0043)$, Sen = $0.9867 (\pm 0.0120)$, and Prec = $0.9809 (\pm 0.0136)$ when reducing the number of descriptors from 7648 to 6400. Furthermore, to classify the Warwick-QU dataset, only 900 (out of 1792 attributes) are needed to achieve the highest accuracy of $96.29\% (\pm 7.89\%$) with AUC = $0.9860 (\pm 0.0353$), Sen = $0.9571 (\pm 0.0964)$, and Prec = $0.9589 (\pm 0.0945)$. For a completion for feature reduction, we provide the confusion matrices of each best performing SVM model while using the reduced set of features as shown in Fig. 8.

**Fig. 9:**
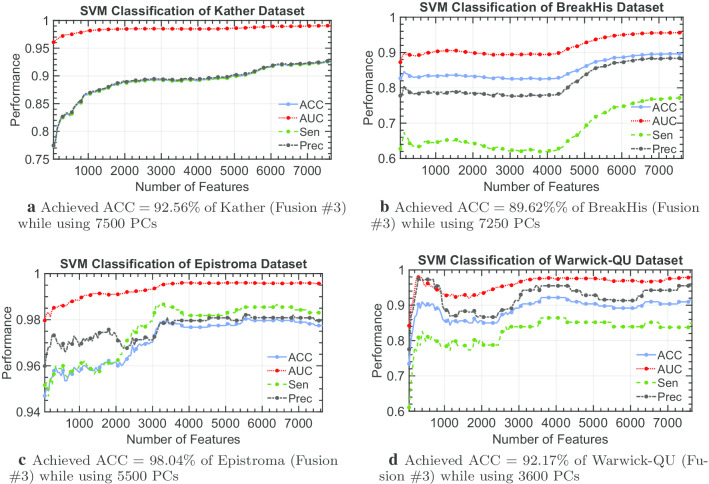
Classification performance of SVM with an increment of 50 PCs of fusion #3 till all PCs are used

Furthermore, the complexity of histopathological images differs from one dataset to another. Although our proposed individual Shearlet-based descriptors are capable of achieving reasonable accuracy across the four histopathological image datasets, the best fusion which can be used to achieve the highest potential accuracy classifying the histological image dataset is not always the same (as shown in Fig. 7). Considering Fusion #3 of our Shearlet-based descriptors expressed in the principal components, we can observe different patterns of classification performance while using an increment of 50 PCs in Fig. 9. Therefore, our Shearlet-based descriptors appear to be general enough for any histological image analysis, and carefully choosing a type of fusion that maximize the recognition of the tissue type is sufficient.

In the literature exist state-of-the-art techniques that use the same datasets that we have used in our study. We report their results in this section to show that our proposed techniques are on par with those techniques and achieve excellent performance when reporting performance in terms of accuracy and AUC. Our Shearlet-based descriptors can attain robust classification performance in different scenarios; hence, it can be an appealing system in the clinical settings for medical image classification. In comparison, Wang et al. [15] proposed to use a bilinear CNN model for classifying multi-class Kather’s dataset. The reported accuracy was 92.6% (and AUC = 0.985). However, our proposed technique can achieve similar accuracy, yet better AUC = 0.9905.

When it comes to the BreakHis dataset, Jonnalagedda et al. [30] trained a CNN model with tumor nuclei information while utilizing data augmentation. Jonnalagedda et al. approach led to an accuracy of 92.2% (and AUC = 0.92). Our approach on BreakHis dataset is efficient to achieve higher AUC value, but with somewhat lower accuracy.

When it comes to Epistroma dataset classification, Ramalho et al. [48] proposed a structural approach using co-occurrence statistics. Their approach led to an accuracy of $\approx$ 95%; our approach attains better classification accuracy.

In the case of Warwick-QU dataset classification, Ribeiro et al. [7] proposed to extract descriptors from the spatial and curvelet domain (i.e., Fractal measures and Haralick features) for which they report a higher AUC of 0.994 but they did not report accuracy.

Evidently, our proposed descriptors compete with the state-of-the-art including those based on CNN in terms of classification accuracy and AUC. In contrast to the resource extensive CNN-based techniques, we hand-engineer our descriptors, yet realize similar results. Our approach is based on different descriptors in the Shaerlet domain which is capable of handling the scarcity of biomedical datasets. The highest accuracies in our study are obtained using an SVM model which is considered as a black box and the classifications made by such models are difficult to interpret and explain [28]. In addition to SVM, we explored DTB which is capable of achieving reasonable results, and might be a more favorable approach in certain applications as they have higher interpretability [49]. However, navigating through a rule to understand a decision made by a DTB is still a challenge since our main descriptors are computed based on Shearlet coefficients. Therefore, linking the computed statistical information from the Shearlet transform into a particular region of interest in a tissue type can be difficult. It is worth noting that our main concern in this study is to establish a robust approach for classifying histological images.

## Conclusion and future work

In this study, we have constructed a novel feature representation for classifying histology tissues. Our technique integrates various sets of textural descriptors which are obtained in the complex Shearlet domain instead of directly computing the descriptors from the gray-level images. Those computed sets of descriptors are based on methods that capture local and global statistics: descriptors from the co-occurrence matrix, local binary patterns, local oriented statistic information booster, and segmentation-based textural features. As a result, we exploit the multi-directionality and multi-resolution of complex Shearlet transform; hence, we investigate the benefits of not only using the magnitude but also the relative phase (RP) components of the complex Shearlet coefficients. We have concluded that in general using both the magnitude and RP can lead to rigorous and effective classification results for histopathological image datasets. We utilize PCA to obtain a reduced set of our proposed integrated feature representation in the Shearlet domain which on some dataset can reduce feature set size while maintaining or increasing classification performance with traditional machine learning. We also show that the machine learning method has only limited influence on the results and hence it is possible to use DTB if desired because decisions of the classifier are considered interpretable. Our proposed method attains state-of-the-art classification results on the four histopathological datasets that we have utilized in this research. Our expectation that our technique is capable to generalize also on other histopathological datasets.

In the future, we plan to further benchmark our proposed techniques with different classification models (e.g., multilayer perceptron, Random Under Sampling Boost decision tree, and others). Additionally, we plan to investigate other feature reduction methodologies to aggressively reduce the number of descriptors without compromising the classification performance. Finally, the problem of a highly imbalanced dataset (i.e., observed in the BreakHis dataset) can be eliminated using sampling methods (e.g., Synthetic minority oversampling (SMOTE) technique) on the training dataset.

## Data Availability

Four publicly available histopathological datasets that have been used in our study. Multi-class Kather’s dataset can be accessed via https://zenodo.org/record/53169/. BreakHis dataset can be accessed via https://web.inf.ufpr.br/vri/databases/breast-cancer-histopathological-database-breakhis/. Epistroma dataset can be accessed via http://fimm.webmicroscope.net/Research/Supplements/epistroma/. Warwick-QU dataset can be accessed via https://warwick.ac.uk/fac/sci/dcs/research/tia/glascontest/download/.

## Appendix

See Tables 1, 2, 3 and 4.

**Table 1:** Classification performance on Kather dataset

Method	Perf	RP + Magnitude		RP		Magnitude	
		SVM	DTB	SVM	DTB	SVM	DTB
*(1): Baseline performance of existing Shearlet-based methods*							
Vo et al.	ACC%	$\underline{82} .\underline{34} \% \pm \underline{1} .\underline{87} \%$	$79.42\% \pm 1.27\%$	$78.46\% \pm 2.11\%$	$75. 22\% \pm 2.17\%$	$71.10\% \pm 1.65\%$	$68.28\% \pm 1.09\%$
	AUC	$\underline{0} .\underline{9658} \pm \underline{0} .\underline{0038}$	$0.9646 \pm 0.0038$	$0.9545 \pm 0.0049$	$0.9552 \pm 0.0066$	$0.9283 \pm 0.0092$	$0.9340 \pm 0.0036$
	Sen	$\underline{0} .\underline{8234} \pm \underline{0} .\underline{0186}$	$0.7943 \pm 0.0127$	$0.7846 \pm 0.0212$	$0.7522 \pm 0.0218$	$0.7111 \pm 0.0161$	$0.6828 \pm 0.0107$
	Prec	$\underline{0} .\underline{8263} \pm \underline{0} .\underline{0191}$	$0.7961 \pm 0.0131$	$0.7858 \pm 0.0218$	$0.7568 \pm 0.0213$	$0.7102 \pm 0.0180$	$0.6826 \pm 0.0131$
Meshkini and Ghassemian	ACC%	$\textit{80.06}\% \pm \textit{1.54}\%$	$\textit{83.06}\% \pm \textit{1.33}\%$	$\textit{37.72}\% \pm \textit{1.78}\%$	$\textit{77.84}\% \pm \textit{1.17}\%$	$83.38\% \pm 1.81\%$	$83.74\% \pm 1.79\%$
	AUC	$\textit{0.9648} \pm \textit{0.0055}$	$\textit{0.9800} \pm \textit{0.0081}$	$\textit{0.7553} \pm \textit{0.0115}$	$\textit{0.9649} \pm \textit{0.0028}$	$0.9738 \pm 0.0036$	$0.9816 \pm 0.0027$
	Sen	$\textit{0.8006} \pm \textit{0.0154}$	$\textit{0.8307} \pm \textit{0.0134}$	$\textit{0.3772} \pm \textit{0.0179}$	$\textit{0.7784} \pm \textit{0.0117}$	$0.8338 \pm 0.0181$	$0.8374 \pm 0.0179$
	Prec	$\textit{0.8111} \pm \textit{0.0128}$	$\textit{0.8325} \pm \textit{0.0124}$	$\textit{0.4338} \pm \textit{0.0193}$	$\textit{0.7792} \pm \textit{0.0105}$	$0.8385 \pm 0.0170$	$0.8400 \pm 0.0168$
Zhou et al.	ACC%	$\textit{64.26}\% \pm \textit{2.20}\%$	$\textit{67.78}\% \pm \textit{1.38}\%$	$\textit{51.34}\% \pm \textit{2.35}\%$	$\textit{54.68}\% \pm \textit{1.79}\%$	$60.06\% \pm 1.43\%$	$64.42\% \pm 1.82\%$
	AUC	$\textit{0.9016} \pm \textit{0.0074}$	$\textit{0.9337} \pm \textit{0.0051}$	$\textit{0.8519} \pm \textit{0.0104}$	$\textit{0.8846} \pm \textit{0.0045}$	$0.8764 \pm 0.0050$	$0.9132 \pm 0.0061$
	Sen	$\textit{0.6425} \pm \textit{0.0222}$	$\textit{0.6778} \pm \textit{0.0138}$	$\textit{0.5134} \pm \textit{0.0233}$	$\textit{0.5468} \pm \textit{0.0177}$	$0.6006 \pm 0.0143$	$0.6442 \pm 0.0183$
	Prec	$\textit{0.6494} \pm \textit{0.0240}$	$\textit{0.6842} \pm \textit{0.0208}$	$\textit{0.5112} \pm \textit{0.0235}$	$\textit{0.5540} \pm \textit{0.0210}$	$0.6182 \pm 0.0198$	$0.6511 \pm 0.0197$
Dong et al.	ACC%	$\textit{81.10}\% \pm \textit{1.90}\%$	$\textit{77.50}\% \pm \textit{1.17}\%$	$\textit{70.66}\% \pm \textit{1.81}\%$	$\textit{72.10}\% \pm \textit{0.97}\%$	$76.62\% \pm 1.86\%$	$72.08\% \pm 1.65\%$
	AUC	$\textit{0.9621} \pm \textit{0.0046}$	$\textit{0.9620} \pm \textit{0.0033}$	$\textit{0.9297} \pm \textit{0.0082}$	$\textit{0.9457} \pm \textit{0.0037}$	$0.9504 \pm 0.0063$	$0.9448 \pm 0.0046$
	Sen	$\textit{0.8110} \pm \textit{0.0188}$	$\textit{0.7751} \pm \textit{0.0116}$	$\textit{0.7066} \pm \textit{0.0179}$	$\textit{0.7210} \pm \textit{0.0100}$	$0.7662 \pm 0.0185$	$0.7207 \pm 0.0166$
	Prec	$\textit{0.8146} \pm \textit{0.0187}$	$\textit{0.7753} \pm \textit{0.0140}$	$\textit{0.7114} \pm \textit{0.0187}$	$\textit{0.7263} \pm \textit{0.0106}$	$0.7681 \pm 0.0190$	$0.7210 \pm 0.0172$
*(2): Proposed Shearlet-based methods for textured bio-medical image classification*							
CM	ACC%	$86.24\% \pm 1.27\%$	$80.98\% \pm 1.54\%$	$82.82\% \pm 1.47\%$	$78.48\% \pm 1.30\%$	$80.28\% \pm 1.64\%$	$76.30\% \pm 1.16\%$
	AUC	$0.9761 \pm 0.0027$	$0.9712 \pm 0.0031$	$0.9697 \pm 0.0035$	$0.9636 \pm 0.0046$	$0.9603 \pm 0.0039$	$0.9568 \pm 0.0051$
	Sen	$0.8624 \pm 0.0128$	$0.8098 \pm 0.0155$	$0.8282 \pm 0.0148$	$0.7849 \pm 0.01274$	$0.8028 \pm 0.0166$	$0.7630 \pm 0.0114$
	Prec	$0.8649 \pm 0.0130$	$0.8127 \pm 0.0141$	$0.8322 \pm 0.0147$	$0.7884 \pm 0.0115$	$0.8037 \pm 0.0157$	$0.7642 \pm 0.0127$
LBP	ACC%	$84.24\% \pm 0.91\%$	$77.90\% \pm 1.75\%$	$81.36\% \pm 1.16\%$	$75.10\% \pm 2.20\%$	$79.36\% \pm 1.39\%$	$75.38\% \pm 1.71\%$
	AUC	$0.9724 \pm 0.0026$	$0.9630 \pm 0.0043$	$0.9649 \pm 0.0039$	$0.9559 \pm 0.0053$	$0.9590 \pm 0.0037$	$0.9541 \pm 0.0064$
	Sen	$0.8424 \pm 0.0092$	$0.7791 \pm 0.0174$	$0.8136 \pm 0.0115$	$0.7511 \pm 0.0216$	$0.7936 \pm 0.0140$	$0.7537 \pm 0.0172$
	Prec	$0.8454 \pm 0.0106$	$0.7829 \pm 0.0185$	$0.8171 \pm 0.0129$	$0.7551 \pm 0.0231$	$0.7964 \pm 0.0131$	$0.7573 \pm 0.0164$
LOSIB	ACC%	$\underline{86} .\underline{32} \% \pm \underline{1} .\underline{36} \%$	$79.78\% \pm 2.11\%$	$82.36\% \pm 1.82\%$	$79.76\% \pm 1.35\%$	$76.52\% \pm 1.69\%$	$71.24\% \pm 1.01\%$
	AUC	$\underline{0} .\underline{9773} \pm \underline{0} .\underline{0021}$	$0.9703 \pm 0.0046$	$0.9671 \pm 0.0045$	$0.9689 \pm 0.0024$	$0.9495 \pm 0.0048$	$0.9402 \pm 0.0037$
	Sen	$\underline{0} .\underline{8632} \pm \underline{0} .\underline{0134}$	$0.7978 \pm 0.0209$	$0.8236 \pm 0.0180$	$0.7976 \pm 0.0136$	$0.7653 \pm 0.0169$	$0.7124 \pm 0.0098$
	Prec	$\underline{0} .\underline{8664} \pm \underline{0} .\underline{0128}$	$0.8017 \pm 0.0216$	$0.8274 \pm 0.0169$	$0.8008 \pm 0.0127$	$0.7672 \pm 0.0191$	$0.7128 \pm 0.0123$
SFTA	ACC%	$82.92\% \pm 1.41\%$	$78.24\% \pm 1.21\%$	$78.10\% \pm 1.43\%$	$75.04\% \pm 1.44\%$	$79.72\% \pm 1.07\%$	$75.42\% \pm 2.23\%$
	AUC	$0.9682 \pm 0.0030$	$0.9645 \pm 0.0035$	$0.9554 \pm 0.0031$	$0.9570 \pm 0.0047$	$0.9597 \pm 0.0032$	$0.9574 \pm 0.0054$
	Sen	$0.8292 \pm 0.0143$	$0.7825 \pm 0.0124$	$0.7810 \pm 0.0141$	$0.7504 \pm 0.0144$	$0.7972 \pm 0.0110$	$0.7543 \pm 0.0224$
	Prec	$0.8331 \pm 0.0139$	$0.7845 \pm 0.0117$	$0.7877 \pm 0.0144$	$0.7539 \pm 0.0133$	$0.7986 \pm 0.0109$	$0.7587 \pm 0.0248$
*(3): Integrating Shearlet-based existing techniques with our proposed methods*							
Fusion #1	ACC%	$87.54\% \pm 1.85\%$	$81.40\% \pm 1.21\%$	$85.32\% \pm 1.72\%$	$80.30\% \pm 1.52\%$	$83.92\% \pm 1.84\%$	$77.76\% \pm 1.21\%$
	AUC	$0.9823 \pm 0.0042$	$0.9734 \pm 0.0019$	$0.9774 \pm 0.0043$	$0.9707 \pm 0.0024$	$0.9740 \pm 0.0049$	$0.9619 \pm 0.0031$
	Sen	$0.8755 \pm 0.0186$	$0.8141 \pm 0.0122$	$0.8533 \pm 0.0173$	$0.8029 \pm 0.0155$	$0.8392 \pm 0.0185$	$0.7776 \pm 0.0121$
	Prec	$0.8764 \pm 0.0189$	$0.8154 \pm 0.0135$	$0.8539 \pm 0.0179$	$0.8078 \pm 0.0153$	$0.8397 \pm 0.0178$	$0.7798 \pm 0.0127$
Fusion #2	ACC%	$88.46\% \pm 1.14\%$	$82.28\% \pm 1.46\%$	$85.14\% \pm 1.05\%$	$80.26\% \pm 2.11\%$	$83.54\% \pm 1.30\%$	$77.20\% \pm 1.92\%$
	AUC	$0.9804 \pm 0.0023$	$0.9746 \pm 0.0034$	$0.9749 \pm 0.0029$	$0.9699 \pm 0.0049$	$0.9693 \pm 0.0033$	$0.9606 \pm 0.0040$
	Sen	$0.8846 \pm 0.0113$	$0.8228 \pm 0.0148$	$0.8514 \pm 0.0105$	$0.8026 \pm 0.0211$	$0.8354 \pm 0.0133$	$0.7720 \pm 0.0195$
	Prec	$0.8874 \pm 0.0108$	$0.8253 \pm 0.0149$	$0.8551 \pm 0.0107$	$0.8070 \pm 0.0198$	$0.8375 \pm 0.0130$	$0.7744 \pm 0.0182$
Fusion #3	ACC%	$\underline{92} .\underline{54} \% \pm \underline{1} .\underline{32} \%$	$88.60\% \pm 2.03\%$	-	-	-	-
	AUC	$\underline{0} .\underline{9906} \pm \underline{0} .\underline{0019}$	$0.9900 \pm 0.0020$	-	-	-	-
	Sen	$\underline{0} .\underline{9254} \pm \underline{0} .\underline{0132}$	$0.8860 \pm 0.0203$	-	-	-	-
	Prec	$\underline{0} .\underline{9267} \pm \underline{0} .\underline{0129}$	$0.8881 \pm 0.0194$	-	-	-	-

The results shown in italic are of experiments that are not explored in the original research papers

The underlined classification results represent the highest results for each corresponding section

**Table 2:** Classification performance on **BreakHis** dataset

Method	Perf	RP + Magnitude		RP		Magnitude	
		SVM	DTB	SVM	DTB	SVM	DTB
*(1): Baseline performance of existing Shearlet-based methods*							
Vo et al.	ACC%	$\underline{85} .\underline{69} \% \pm \underline{1} .\underline{18} \%$	$80.65 \% \pm 1.05\%$	$83.63\% \pm 0.99\%$	$78.95\% \pm 0.74\%$	$76.10\% \pm 0.66\%$	$76.52\% \pm 0.64\%$
	AUC	$\underline{0} .\underline{9121} \pm \underline{0} .\underline{0053}$	$0.8687 \pm 0.0079$	$0.8915 \pm 0.0103$	$0.8443 \pm 0.0183$	$0.7717 \pm 0.0123$	$0.7926 \pm 0.0175$
	Sen	$\underline{0} .\underline{7210} \pm \underline{0} .\underline{0276}$	$0.4855 \pm 0.0346$	$0.6766 \pm 0.0231$	$0.4496 \pm 0.0157$	$0.4698 \pm 0.0213$	$0.4056 \pm 0.0301$
	Prec	$\underline{0} .\underline{8035} \pm \underline{0} .\underline{0301}$	$0.8268 \pm 0.0296$	$0.7732 \pm 0.0201$	$0.7881 \pm 0.0208$	$0.6701 \pm 0.0192$	$0.7247 \pm 0.0159$
Meshkini and Ghassemian	ACC%	$\textit{78.06}\% \pm \textit{0.80}\%$	$\textit{79.43} \% \pm \textit{1.09}\%$	$\textit{77.33}\% \pm \textit{1.23}\%$	$\textit{79.76}\% \pm \textit{0.71}\%$	$76.44\% \pm 0.96\%$	$77.46\% \pm 0.85\%$
	AUC	$\textit{0.8339} \pm \textit{0.0030}$	$\textit{0.8523} \pm \textit{0.0055}$	$\textit{0.8111} \pm \textit{0.0086}$	$\textit{0.8579} \pm \textit{0.0078}$	$0.7991 \pm 0.0139$	$0.8357 \pm 0.0112$
	Sen	$\textit{0.5177} \pm \textit{0.0194}$	$\textit{0.4927} \pm \textit{0.0213}$	$\textit{0.4899} \pm \textit{0.0291}$	$\textit{0.4980} \pm \textit{0.0237}$	$0.4722 \pm 0.0232$	$0.4218 \pm 0.0246$
	Prec	$\textit{0.7049} \pm \textit{0.0223}$	$\textit{0.7688} \pm \textit{0.0313}$	$\textit{0.6973} \pm \textit{0.0284}$	$\textit{0.7779} \pm \textit{0.0300}$	$0.6788 \pm 0.0215$	$0.7496 \pm 0.0181$
Zhou et al.	ACC%	$\textit{68.66}\% \pm \textit{1.23}\%$	$\textit{70.21} \% \pm \textit{0.36}\%$	$\textit{65.20}\% \pm \textit{1.47}\%$	$\textit{69.64}\% \pm \textit{0.47}\%$	$66.47\% \pm 0.88\%$	$70.58\% \pm 0.49\%$
	AUC	$\textit{0.6426} \pm \textit{0.0245}$	$\textit{0.6781} \pm \textit{0.0173}$	$\textit{0.5832} \pm \textit{0.0193}$	$\textit{0.6154} \pm \textit{0.0116}$	$0.6022 \pm 0.0198$	$0.6709 \pm 0.0165$
	Sen	$\textit{0.2875} \pm \textit{0.0310}$	$\textit{0.0677} \pm \textit{0.0139}$	$\textit{0.2290} \pm \textit{0.0273}$	$\textit{0.0456} \pm \textit{0.0096}$	$0.2415 \pm 0.0326$	$0.0956 \pm 0.0123$
	Prec	$\textit{0.4996} \pm \textit{0.0357}$	$\textit{0.7915} \pm \textit{0.0314}$	$\textit{0.4035} \pm \textit{0.0391}$	$\textit{0.7728} \pm \textit{0.1106}$	$0.4361 \pm 0.0291$	$0.7436 \pm 0.0687$
Dong et al.	ACC%	$\textit{79.49}\% \pm \textit{1.39}\%$	$\textit{78.40} \% \pm \textit{0.54}\%$	$\textit{73.54}\% \pm \textit{1.61}\%$	$\textit{75.58}\% \pm \textit{0.78}\%$	$78.40\ \pm 1.20\%$	$77.56\% \pm 0.97\%$
	AUC	$\textit{0.8390} \pm \textit{0.0163}$	$\textit{0.8359} \pm \textit{0.0110}$	$\textit{0.7641} \pm \textit{0.0172}$	$\textit{0.8018} \pm \textit{0.0069}$	$0.8090 \pm 0.0114$	$0.8082 \pm 0.0156$
	Sen	$\textit{0.5980} \pm \textit{0.0342}$	$\textit{0.4360} \pm \textit{0.0139}$	$\textit{0.4860} \pm \textit{0.0312}$	$\textit{0.3328} \pm \textit{0.0327}$	$0.5176 \pm 0.0270$	$0.4380 \pm 0.0129$
	Prec	$\textit{0.7077} \pm \textit{0.0226}$	$\textit{0.7855} \pm \textit{0.0169}$	$\textit{0.6011} \pm \textit{0.0324}$	$\textit{0.7600} \pm \textit{0.0155}$	$0.7204 \pm 0.0243$	$0.7480 \pm 0.0307$
*(2): Proposed Shearlet-based methods for textured bio-medical image classification*							
CM	ACC%	$87.26\% \pm 1.18\%$	$79.33 \% \pm 1.17\%$	$86.93\% \pm 0.75\%$	$79.95\% \pm 0.39\%$	$78.85\% \pm 1.42\%$	$75.08\% \pm 0.80\%$
	AUC	$0.9365 \pm 0.0104$	$0.8679 \pm 0.0134$	$0.9283 \pm 0.0052$	$0.8687 \pm 0.0085$	$0.8350 \pm 0.0143$	$0.7823 \pm 0.0148$
	Sen	$0.7391 \pm 0.0286$	$0.4137 \pm 0.0351$	$0.7520 \pm 0.0242$	$0.4677 \pm 0.0188$	$0.5649 \pm 0.0371$	$0.2911 \pm 0.0208$
	Prec	$0.8358 \pm 0.0230$	$0.8500 \pm 0.0270$	$0.8166 \pm 0.0108$	$0.8140 \pm 0.0164$	$0.7021 \pm 0.0253$	$0.7717 \pm 0.0274$
LBP	ACC%	$89.51\% \pm 0.69\%$	$81.63 \% \pm 0.96\%$	$87.15\% \pm 0.51\%$	$79.58\% \pm 1.05\%$	$86.07\% \pm 1.04\%$	$80.01\% \pm 1.11\%$
	AUC	$0.9477 \pm 0.0043$	$0.8905 \pm 0.0101$	$0.9296 \pm 0.0054$	$0.8665 \pm 0.0131$	$0.9153 \pm 0.0080$	$0.8630 \pm 0.0075$
	Sen	$0.7964 \pm 0.0233$	$0.5081 \pm 0.0180$	$0.7508 \pm 0.0173$	$0.4484 \pm 0.0305$	$0.7177 \pm 0.0355$	$0.4641 \pm 0.0296$
	Prec	$0.8590 \pm 0.0149$	$0.8444 \pm 0.0299$	$0.8241 \pm 0.0126$	$0.8182 \pm 0.0212$	$0.8158 \pm 0.0132$	$0.8200 \pm 0.0219$
LOSIB	ACC%	$87.81\% \pm 0.63\%$	$80.63\% \pm 1.03\%$	$85.78 \% \pm 0.52\%$	$78.94\% \pm 1.47\%$	$80.31\% \pm 1.09\%$	$78.49\% \pm 0.90\%$
	AUC	$0.9295 \pm 0.0056$	$0.8771 \pm 0.0073$	$0.9092 \pm 0.0042$	$0.8516 \pm 0.0174$	$0.8486 \pm 0.0085$	$0.8206 \pm 0.0119$
	Sen	$0.7585 \pm 0.0252$	$0.4972 \pm 0.0225$	$0.7234 \pm 0.0177$	$0.4315 \pm 0.0284$	$0.5665 \pm 0.0274$	$0.4617 \pm 0.0170$
	Prec	$0.8380 \pm 0.0163$	$0.8121 \pm 0.0220$	$0.8036 \pm 0.0116$	$0.8071 \pm 0.0436$	$0.7447 \pm 0.0220$	$0.7580 \pm 0.0247$
SFTA	ACC%	$\underline{89} .\underline{72} \% \pm \underline{0} .\underline{63} \%$	$81.83\% \pm 0.79\%$	$87.95\% \pm 0.57\%$	$79.90\% \pm 1.20\%$	$83.53\% \pm 1.04\%$	$80.39\% \pm 1.14\%$
	AUC	$\underline{0} .\underline{9527} \pm \underline{0} .\underline{0055}$	$0.8880 \pm 0.0071$	$0.9393 \pm 0.0065$	$0.8769 \pm 0.0070$	$0.8943 \pm 0.0090$	$0.8424 \pm 0.0158$
	Sen	$\underline{0} .\underline{8040} \pm \underline{0} .\underline{0243}$	$0.4992 \pm 0.0265$	$0.7701 \pm 0.0239$	$0.4601 \pm 0.0251$	$0.6645 \pm 0.0332$	$0.4665 \pm 0.0220$
	Prec	$\underline{0} .\underline{8593} \pm \underline{0} .\underline{0112}$	$0.8641 \pm 0.0137$	$0.8332 \pm 0.0112$	$0.8197 \pm 0.0324$	$0.7787 \pm 0.0264$	$0.8355 \pm 0.0323$
*(3): Integrating Shearlet-based existing techniques with our proposed methods*							
Fusion #1	ACC%	$\underline{91} .\underline{28} \% \pm \underline{0} .\underline{51} \%$	$81.78\% \pm 0.73\%$	$89.58\% \pm 0.83\%$	$80.98\% \pm 0.71\%$	$87.29\% \pm 0.37\%$	$80.30\% \pm 0.62\%$
	AUC	$\underline{0} .\underline{9650} \pm \underline{0} .\underline{0031}$	$0.8981 \pm 0.0053$	$0.9515 \pm 0.0031$	$0.8853 \pm 0.0102$	$0.9346 \pm 0.0051$	$0.8719 \pm 0.0084$
	Sen	$\underline{0} .\underline{8391} \pm \underline{0} .\underline{0147}$	$0.5085 \pm 0.0087$	$0.8121 \pm 0.0119$	$0.4855 \pm 0.0252$	$0.7561 \pm 0.0214$	$0.4504 \pm 0.0163$
	Prec	$\underline{0} .\underline{8775} \pm \underline{0} .\underline{0120}$	$0.8508 \pm 0.0259$	$0.8495 \pm 0.0219$	$0.8412 \pm 0.0185$	$0.8244 \pm 0.0094$	$0.8516 \pm 0.0198$
Fusion #2	ACC%	$89.33\% \pm 0.61\%$	$80.33\% \pm 0.89\%$	$87.82\% \pm 0.36\%$	$80.24\% \pm 0.84\%$	$83.23\% \pm 0.93\%$	$77.80\% \pm 0.51\%$
	AUC	$0.9503 \pm 0.0053$	$0.8763 \pm 0.0138$	$0.9354 \pm 0.0037$	$0.8742 \pm 0.0072$	$0.8872 \pm 0.0098$	$0.8446 \pm 0.0071$
	Sen	$0.7964 \pm 0.0245$	$0.4544 \pm 0.0202$	$0.7690 \pm 0.0132$	$0.4726 \pm 0.0171$	$0.6536 \pm 0.0155$	$0.3694 \pm 0.0173$
	Prec	$0.8537 \pm 0.0105$	$0.8471 \pm 0.0213$	$0.8304 \pm 0.0098$	$0.8216 \pm 0.0252$	$0.7770 \pm 0.0256$	$0.8270 \pm 0.0170$
Fusion #3	ACC%	$90.10\% \pm 0.87\%$	$85.08\% \pm 0.80\%$	-	-	-	-
	AUC	$0.9560 \pm 0.0053$	$0.9256 \pm 0.0065$	-	-	-	-
	Sen	$0.8028 \pm 0.0113$	$0.5992 \pm 0.0175$	-	-	-	-
	Prec	$0.8723 \pm 0.0281$	$0.8887 \pm 0.0161$	-	-	-	-

The results shown in italic are of experiments that are not explored in the original research papers

The underlined classification results represent the highest results for each corresponding section

**Table 3:** Classification performance on **Epistroma** dataset

Method	Perf	RP + Magnitude		RP		Magnitude	
		SVM	DTB	SVM	DTB	SVM	DTB
*(1): Baseline performance of existing Shearlet-based methods*							
Vo et al.	ACC%	$\underline{96} .\underline{44} \% \pm \underline{1} .\underline{47} \%$	$95.42\% \pm 1.03\%$	$95.50\% \pm 1.52\%$	$94.26\% \pm 1.95\%$	$94.48\% \pm 1.54\%$	$94.26\% \pm 1.35\%$
	AUC	$\underline{0} .\underline{9895} \pm \underline{0} .\underline{0082}$	$0.9897 \pm 0.0047$	$0.9873 \pm 0.0078$	$0.9839 \pm 0.0087$	$0.9830 \pm 0.0083$	$0.9763 \pm 0.0102$
	Sen	$\underline{0} .\underline{9685} \pm \underline{0} .\underline{0131}$	$0.9443 \pm 0.0162$	$0.9685 \pm 0.0117$	$0.9467 \pm 0.0268$	$0.9588 \pm 0.0182$	$0.9564 \pm 0.0152$
	Prec	$\underline{0} .\underline{9722} \pm \underline{0} .\underline{0170}$	$0.9787 \pm 0.0083$	$0.9577 \pm 0.0260$	$0.9576 \pm 0.0220$	$0.9501\pm 0.0219$	$0.9487 \pm 0.0193$
Meshkini and Ghassemian	ACC%	$\textit{96.08}\% \pm \textit{1.24}\%$	$\textit{96.29}\% \pm \textit{1.86}\%$	$\textit{95.35}\% \pm \textit{1.37}\%$	$\textit{96.37} \% \pm \textit{2.30}\%$	$95.35\% \pm 1.49\%$	$96.44 \% \pm 2.20\%$
	AUC	$\textit{0.9926} \pm \textit{0.0046}$	$\textit{0.9929} \pm \textit{0.0041}$	$\textit{0.9887} \pm \textit{0.0058}$	$\textit{0.9919} \pm \textit{0.0077}$	$0.9899 \pm 0.0050$	$0.9908 \pm 0.0068$
	Sen	$\textit{0.9710} \pm \textit{0.0190}$	$\textit{0.9600} \pm \textit{0.0222}$	$\textit{0.9685} \pm \textit{0.0207}$	$\textit{0.9600} \pm \textit{0.0269}$	$0.9722 \pm 0.0161$	$0.9564 \pm 0.0274$
	Prec	$\textit{0.9642} \pm \textit{0.0169}$	$\textit{0.9778} \pm \textit{0.0151}$	$\textit{0.9550} \pm \textit{0.0183}$	$\textit{0.9791} \pm \textit{0.0184}$	$0.9517 \pm 0.0186$	$0.9840 \pm 0.0181$
Zhou et al.	ACC%	$\textit{89.68}\% \pm \textit{1.33}\%$	$\textit{88.30}\% \pm \textit{2.68}\%$	$\textit{81.69}\% \pm \textit{2.53}\%$	$\textit{79.65} \% \pm \textit{3.11}\%$	$84.45\% \pm 2.98\%$	$87.13 \% \pm 2.95\%$
	AUC	$\textit{0.9506} \pm \textit{0.0158}$	$\textit{0.9400} \pm \textit{0.0223}$	$\textit{0.8748} \pm \textit{0.0222}$	$\textit{0.8721} \pm \textit{0.0367}$	$0.9110 \pm 0.0194$	$0.9416 \pm 0.0244$
	Sen	$\textit{0.9370} \pm \textit{0.0219}$	$\textit{0.9103} \pm \textit{0.0287}$	$\textit{0.8934} \pm \textit{0.0363}$	$\textit{0.8666} \pm \textit{0.0343}$	$0.9236 \pm 0.0141$	$0.9102 \pm 0.0327$
	Prec	$\textit{0.8961} \pm \textit{0.0183}$	$\textit{0.8968} \pm \textit{0.0275}$	$\textit{0.8186} \pm \textit{0.0251}$	$\textit{0.8088} \pm \textit{0.0301}$	$0.8357 \pm 0.0330$	$0.8799 \pm 0.0282$
Dong et al.	ACC%	$\textit{95.72}\% \pm \textit{1.54}\%$	$\textit{95.28}\% \pm \textit{1.66}\%$	$\textit{94.84}\% \pm \textit{1.59}\%$	$\textit{93.39}\% \pm \textit{2.11}\%$	$95.86\% \pm 1.32\%$	$93.75\% \pm 1.15\%$
	AUC	$\textit{0.9882} \pm \textit{0.0071}$	$\textit{0.9837} \pm \textit{0.0089}$	$\textit{0.9836} \pm \textit{0.0071}$	$\textit{0.9776} \pm \textit{0.0132}$	$0.9884 \pm 0.0082$	$0.9836 \pm 0.0101$
	Sen	$\textit{0.9551} \pm \textit{0.0162}$	$\textit{0.9503} \pm \textit{0.0271}$	$\textit{0.9503} \pm \textit{0.0218}$	$\textit{0.9187} \pm \textit{0.0300}$	$0.9685 \pm 0.0103$	$0.9600 \pm 0.0182$
	Prec	$\textit{0.9735} \pm \textit{0.0227}$	$\textit{0.9707} \pm \textit{0.0178}$	$\textit{0.9637} \pm \textit{0.0211}$	$\textit{0.9697} \pm \textit{0.0201}$	$0.9630 \pm 0.0194$	$0.9381 \pm 0.0231$
*(2): Proposed Shearlet-Based Methods For Textured Bio-medical Image Classification*							
CM	ACC%	$\underline{97} .\underline{24} \% \pm \underline{1} .\underline{27} \%$	$94.41\% \pm 1.48\%$	$96.66\% \pm 1.14\%$	$94.99\% \pm 1.43\%$	$96.00\% \pm 1.30\%$	$94.19\% \pm 2.19\%$
	AUC	$\underline{0} .\underline{9917} \pm \underline{0} .\underline{0067}$	$0.9819 \pm 0.0126$	$0.9925 \pm 0.0064$	$0.9828 \pm 0.0116$	$0.9863 \pm 0.0087$	$0.9828 \pm 0.0091$
	Sen	$\underline{0} .\underline{9733} \pm \underline{0} .\underline{0126}$	$0.9419 \pm 0.0258$	$0.9672 \pm 0.0129$	$0.9503 \pm 0.0134$	$0.9636 \pm 0.0163$	$0.9418 \pm 0.0294$
	Prec	$\underline{0} .\underline{9807} \pm \underline{0} .\underline{0150}$	$0.9646 \pm 0.0202$	$0.9769 \pm 0.0130$	$0.9658 \pm 0.0175$	$0.9696 \pm 0.0116$	$0.9607 \pm 0.0186$
LBP	ACC%	$95.64\% \pm 1.32\%$	$95.57\% \pm 2.04\%$	$95.50\% \pm 1.06\%$	$94.04\% \pm 1.56\%$	$95.71\% \pm 1.39\%$	$93.90\% \pm 1.75\%$
	AUC	$0.9890 \pm 0.0073$	$0.9905 \pm 0.0069$	$0.9871 \pm 0.0076$	$0.9850 \pm 0.0054$	$0.9876 \pm 0.0088$	$0.9800 \pm 0.0113$
	Sen	$0.9624 \pm 0.0146$	$0.9479 \pm 0.0261$	$0.9661 \pm 0.0178$	$0.9394 \pm 0.0221$	$0.9600 \pm 0.0153$	$0.9406 \pm 0.0239$
	Prec	$0.9653 \pm 0.0203$	$0.9779 \pm 0.0210$	$0.9594 \pm 0.0133$	$0.9607 \pm 0.0176$	$0.9687 \pm 0.0206$	$0.9573 \pm 0.0194$
LOSIB	ACC%	$96.29\% \pm 1.47\%$	$95.13\% \pm 1.61\%$	$95.50\% \pm 1.32\%$	$96.65\% \pm 1.51\%$	$95.35\% \pm 1.85\%$	$93.10\% \pm 1.29\%$
	AUC	$0.9900 \pm 0.0069$	$0.9870 \pm 0.0074$	$0.9868 \pm 0.0073$	$0.9911 \pm 0.0065$	$0.9892 \pm 0.0087$	$0.9825 \pm 0.0103$
	Sen	$0.9733 \pm 0.0096$	$0.9454 \pm 0.0245$	$0.9721 \pm 0.0152$	$0.9624 \pm 0.0218$	$0.9758 \pm 0.0141$	$0.9503 \pm 0.0276$
	Prec	$0.9656 \pm 0.0215$	$0.9729 \pm 0.0179$	$0.9541 \pm 0.0203$	$0.9816 \pm 0.0132$	$0.9487 \pm 0.0239$	$0.9362 \pm 0.0194$
SFTA	ACC%	$95.71\% \pm 1.54\%$	$93.90\% \pm 1.81\%$	$95.28\% \pm 2.16\%$	$91.57\% \pm 1.91\%$	$94.33\% \pm 1.52\%$	$92.01 \% \pm 2.07\%$
	AUC	$0.9881 \pm 0.0064$	$0.9841 \pm 0.0106$	$0.9870 \pm 0.0068$	$0.9775 \pm 0.0093$	$0.9824 \pm 0.0080$	$0.9732 \pm 0.0125$
	Sen	$0.9661 \pm 0.0137$	$0.9297 \pm 0.0282$	$0.9624 \pm 0.0145$	$0.9006 \pm 0.0265$	$0.9636 \pm 0.0114$	$0.9455 \pm 0.0221$
	Prec	$0.9628 \pm 0.0169$	$0.9676 \pm 0.0184$	$0.9593 \pm 0.0233$	$0.9572 \pm 0.0266$	$0.9438 \pm 0.024$	$0.9232 \pm 0.0178$
*(3): Integrating Shearlet-based existing techniques with our proposed methods*							
Fusion #1	ACC%	$96.59\% \pm 1.24\%$	$95.93\% \pm 1.95\%$	$96.37\% \pm 1.14\%$	$94.98\% \pm 0.73\%$	$95.71\% \pm 1.25\%$	$95.13 \% \pm 1.37\%$
	AUC	$0.9889 \pm 0.0111$	$0.9910 \pm 0.0070$	$0.9889 \pm 0.0103$	$0.9838 \pm 0.0131$	$0.9877 \pm 0.0104$	$0.9841 \pm 0.0104$
	Sen	$0.9612 \pm 0.0126$	$0.9624 \pm 0.0224$	$0.9612 \pm 0.0179$	$0.9406 \pm 0.0194$	$0.9563 \pm 0.0155$	$0.9503 \pm 0.0201$
	Prec	$0.9816 \pm 0.0140$	$0.9696 \pm 0.0175$	$0.9780 \pm 0.0135$	$0.9752 \pm 0.0139$	$0.9721 \pm 0.0185$	$0.9682 \pm 0.0172$
Fusion #2	ACC%	$97.09\% \pm 1.49\%$	$94.98\% \pm 1.56\%$	$96.37\% \pm 1.45\%$	$95.86\% \pm 2.11\%$	$96.73\% \pm 1.33\%$	$95.28 \% \pm 1.54\%$
	AUC	$0.9910 \pm 0.0099$	$0.9829 \pm 0.0111$	$0.9908 \pm 0.0092$	$0.9884 \pm 0.0113$	$0.9892 \pm 0.0097$	$0.9872 \pm 0.0106$
	Sen	$0.9660 \pm 0.0205$	$0.9382 \pm 0.0211$	$0.9600 \pm 0.0182$	$0.9527 \pm 0.0271$	$0.9685 \pm 0.0192$	$0.9612 \pm 0.0124$
	Prec	$0.9853 \pm 0.0111$	$0.9778 \pm 0.0213$	$0.9791 \pm 0.0128$	$0.9779 \pm 0.0201$	$0.9771 \pm 0.0158$	$0.9607 \pm 0.0250$
Fusion #3	ACC%	$\underline{97} .\underline{46} \% \pm \underline{1} .\underline{24} \%$	$96.22\% \pm 1.60\%$	-	-	-	-
	AUC	$\underline{0} .\underline{9925} \pm \underline{0} .\underline{0093}$	$0.9920 \pm 0.0047$	-	-	-	-
	Sen	$\underline{0} .\underline{9769} \pm \underline{0} .\underline{0156}$	$0.9551 \pm 0.0229$	-	-	-	-
	Prec	$\underline{0} .\underline{9809} \pm \underline{0} .\underline{0165}$	$0.9814 \pm 0.0103$	-	-	-	-

The results shown in italic are of experiments that are not explored in the original research papers

The underlined classification results represent the highest results for each corresponding section

**Table 4:** Classification performance on **Warwick-QU** dataset

Method	Perf	RP + Magnitude		RP		Magnitude	
		SVM	DTB	SVM	DTB	SVM	DTB
*(1): Baseline performance of existing Shearlet-based methods*							
Vo et al.	ACC%	$\underline{93} .\underline{35} \% \pm \underline{5} .\underline{23} \%$	$86.14\% \pm 6.10\%$	$92.13\% \pm 8.53\%$	$85.55\% \pm 6.83\%$	$73.31\% \pm 11.27\%$	$73.97 \% \pm 12.26\%$
	AUC	$\underline{0} .\underline{9802} \pm \underline{0} .\underline{0351}$	$0.9362 \pm 0.0347$	$0.9829 \pm 0.0345$	$0.9338 \pm 0.0437$	$0.8157 \pm 0.0998$	$0.8163 \pm 0.1047$
	Sen	$\underline{0} .\underline{8929} \pm \underline{0} .\underline{1349}$	$0.8804 \pm 0.1107$	$0.8661 \pm 0.1661$	$0.8018 \pm 0.1201$	$0.6750 \pm 0.1976$	$0.6625 \pm 0.1872$
	Prec	$\underline{0} .\underline{9639} \pm \underline{0} .\underline{0583}$	$0.8294 \pm 0.0786$	$0.9579 \pm 0.0690$	$0.8696 \pm 0.0766$	$0.7256 \pm 0.1569$	$0.7324 \pm 0.1391$
Meshkini and Ghassemian	ACC%	$\textit{82.54}\% \pm \textit{8.52}\%$	$\textit{87.90}\% \pm \textit{6.25}\%$	$\textit{86.14}\% \pm \textit{7.25}\%$	$\textit{84.23}\% \pm \textit{9.54}\%$	$81.95\% \pm 8.73\%$	$84.38 \% \pm 8.86\%$
	AUC	$\textit{0.9462} \pm \textit{0.0326}$	$\textit{0.9331} \pm \textit{0.0592}$	$\textit{0.9293} \pm \textit{0.0512}$	$\textit{0.8800} \pm \textit{0.0676}$	$0.9013 \pm 0.0455$	$0.9329 \pm 0.0546$
	Sen	$\textit{0.7214} \pm \textit{0.1475}$	$\textit{0.7821} \pm \textit{0.1380}$	$\textit{0.7589} \pm \textit{0.1892}$	$\textit{0.7286} \pm \textit{0.1828}$	$0.7196 \pm 0.1409$	$0.8196 \pm 0.1896$
	Prec	$\textit{0.8707} \pm \textit{0.0937}$	$\textit{0.9389} \pm \textit{0.0811}$	$\textit{0.9324} \pm \textit{0.0921}$	$\textit{0.8973} \pm \textit{0.1164}$	$0.8574 \pm 0.1125$	$0.8519 \pm 0.1161$
Zhou et al.	ACC%	$\textit{63.60}\% \pm \textit{7.56}\%$	$\textit{75.85}\% \pm \textit{8.72}\%$	$\textit{55.15}\% \pm \textit{8.52}\%$	$\textit{67.76}\% \pm \textit{10.21}\%$	$58.79\% \pm 6.61\%$	$72.21 \% \pm 9.57\%$
	AUC	$\textit{0.6752} \pm \textit{0.1375}$	$\textit{0.8544} \pm \textit{0.1017}$	$\textit{0.5771} \pm \textit{0.1091}$	$\textit{0.6769} \pm \textit{0.1280}$	$0.6006 \pm 0.1252$	$0.7699 \pm 0.1103$
	Sen	$\textit{0.4571} \pm \textit{0.1358}$	$\textit{0.5964} \pm \textit{0.1550}$	$\textit{0.4054} \pm \textit{0.1296}$	$\textit{0.4804} \pm \textit{0.1944}$	$0.3375 \pm 0.1814$	$0.5821 \pm 0.1092$
	Prec	$\textit{0.6442} \pm \textit{0.1660}$	$\textit{0.8349} \pm \textit{0.1336}$	$\textit{0.5157} \pm \textit{0.1430}$	$\textit{0.6812} \pm \textit{0.1553}$	$0.5764 \pm 0.1998$	$0.7665 \pm 0.1601$
Dong et al.	ACC%	$\textit{91.58}\% \pm \textit{6.33}\%$	$\textit{86.07}\% \pm \textit{11.08}\%$	$\textit{79.96}\% \pm \textit{6.46}\%$	$\textit{83.57}\% \pm \textit{12.54}\%$	$83.13\% \pm 5.94\%$	$75.74\% \pm 5.06\%$
	AUC	$\textit{0.9777} \pm \textit{0.0372}$	$\textit{0.9345} \pm \textit{0.0743}$	$\textit{0.9234} \pm \textit{0.0547}$	$\textit{0.9297} \pm \textit{0.0829}$	$0.9342 \pm 0.0507$	$0.8575 \pm 0.0826$
	Sen	$\textit{0.9589} \pm \textit{0.0663}$	$\textit{0.8125} \pm \textit{0.1831}$	$\textit{0.8089} \pm \textit{0.1007}$	$\textit{0.7536} \pm \textit{0.2074}$	$0.8143 \pm 0.1381$	$0.6625 \pm 0.1112$
	Prec	$\textit{0.8844} \pm \textit{0.1172}$	$\textit{0.8661} \pm \textit{0.1076}$	$0.7673 \pm 0.0976$	$\textit{0.8843} \pm \textit{0.1575}$	$0.8252 \pm 0.0942$	$0.7731 \pm 0.0968$
*(2): Proposed Shearlet-based methods for textured bio-medical image classification*							
CM	ACC%	$\underline{95} .\underline{70} \% \pm \underline{7} .\underline{80} \%$	$86.76\% \pm 5.35\%$	$92.61\% \pm 8.25\%$	$84.82\% \pm 8.92\%$	$85.40\% \pm 6.56\%$	$78.20\% \pm 13.74\%$
	AUC	$\underline{0} .\underline{9860} \pm \underline{0} .\underline{0353}$	$0.9427 \pm 0.0347$	$0.9709 \pm 0.0457$	$0.9372 \pm 0.0451$	$0.9440 \pm 0.0462$	$0.8992 \pm 0.0653$
	Sen	$\underline{0} .\underline{9446} \pm \underline{0} .\underline{0983}$	$0.8679 \pm 0.1226$	$0.9304 \pm 0.0736$	$0.7946 \pm 0.1404$	$0.7946 \pm 0.0782$	$0.7214 \pm 0.2090$
	Prec	$\underline{0} .\underline{9589} \pm \underline{0} .\underline{0945}$	$0.8483\pm 0.0682$	$0.9181 \pm 0.1222$	$0.8691 \pm 0.1277$	$0.8708 \pm 0.1060$	$0.7756 \pm 0.1485$
LBP	ACC%	$88.49\% \pm 5.25\%$	$86.69\% \pm 7.24\%$	$84.12\% \pm 7.49\%$	$80.00\% \pm 11.99\%$	$89.71\% \pm 9.45\%$	$82.43\% \pm 8.30\%$
	AUC	$0.9592 \pm 0.0544$	$0.9418 \pm 0.0416$	$0.9500 \pm 0.0670$	$0.8944 \pm 0.0836$	$0.9419 \pm 0.0538$	$0.9248 \pm 0.0388$
	Sen	$0.8821 \pm 0.1152$	$0.7982 \pm 0.1223$	$0.8518 \pm 0.1021$	$0.7625 \pm 0.1762$	$0.8946 \pm 0.1305$	$0.7304 \pm 0.1685$
	Prec	$0.8770 \pm 0.0955$	$0.9000 \pm 0.0905$	$0.8292 \pm 0.1426$	$0.7921 \pm 0.1434$	$0.8825 \pm 0.1080$	$0.8649 \pm 0.1017$
LOSIB	ACC%	$92.57\% \pm 9.24\%$	$87.21\% \pm 5.39\%$	$89.52\% \pm 7.85\%$	$87.43\% \pm 10.53\%$	$79.34\% \pm 5.33\%$	$76.80\% \pm 10.66\%$
	AUC	$0.9798 \pm 0.0395$	$0.9346 \pm 0.0432$	$0.9671 \pm 0.0554$	$0.9344 \pm 0.0570$	$0.9153 \pm 0.0601$	$0.8679 \pm 0.0907$
	Sen	$0.9446 \pm 0.0983$	$0.7964 \pm 0.1155$	$0.8464 \pm 0.1258$	$0.8411 \pm 0.1442$	$0.7732 \pm 0.1465$	$0.7125 \pm 0.1685$
	Prec	$0.9042 \pm 0.1255$	$0.9177 \pm 0.0902$	$0.9264 \pm 0.1119$	$0.8792 \pm 0.1265$	$0.7983 \pm 0.1328$	$0.7579 \pm 0.1123$
SFTA	ACC%	$94.52\% \pm 7.47\%$	$87.76\% \pm 5.95\%$	$92.68\% \pm 7.06\%$	$84.23\% \pm 10.17\%$	$85.99\% \pm 7.09\%$	$82.39\% \pm 6.18\%$
	AUC	$0.9846 \pm 0.0350$	$0.9353 \pm 0.0469$	$0.9739 \pm 0.0416$	$0.9192 \pm 0.0937$	$0.9515 \pm 0.0410$	$0.9119 \pm 0.0692$
	Sen	$0.9196 \pm 0.0968$	$0.8196 \pm 0.1576$	$0.9179 \pm 0.0710$	$0.7821 \pm 0.1301$	$0.7946 \pm 0.0979$	$0.8286 \pm 0.1203$
	Prec	$0.9589 \pm 0.0945$	$0.9107 \pm 0.0869$	$0.9246 \pm 0.1059$	$0.8767 \pm 0.1447$	$0.8798 \pm 0.0881$	$0.8186 \pm 0.1372$
*(3): Integrating Shearlet-based existing techniques with our proposed methods*							
Fusion #1	ACC%	$95.11\% \pm 7.67\%$	$88.42\% \pm 7.84\%$	$92.65\% \pm 8.26\%$	$92.06\% \pm 7.23\%$	$87.83\% \pm 5.07\%$	$79.45\% \pm 10.88\%$
	AUC	$0.9860 \pm 0.0353$	$0.9527 \pm 0.0475$	$0.9721 \pm 0.0511$	$0.9645 \pm 0.0603$	$0.9651 \pm 0.0302$	$0.8867 \pm 0.0704$
	Sen	$0.9321 \pm 0.0985$	$0.8750 \pm 0.0807$	$0.9321 \pm 0.0718$	$0.9196 \pm 0.0968$	$0.8661 \pm 0.0597$	$0.7429 \pm 0.1543$
	Prec	$0.9589 \pm 0.0945$	$0.8833 \pm 0.1277$	$0.9181 \pm 0.1222$	$0.9125 \pm 0.1006$	$0.8724 \pm 0.0991$	$0.8111 \pm 0.1621$
Fusion #2	ACC%	$\underline{96} .\underline{29} \% \pm \underline{7} .\underline{89} \%$	$90.99\% \pm 7.13\%$	$92.02\% \pm 7.86\%$	$90.81\% \pm 8.42\%$	$87.79\% \pm 5.90\%$	$79.26\% \pm 8.62\%$
	AUC	$\underline{0} .\underline{9860} \pm \underline{0} .\underline{0353}$	$0.9580 \pm 0.0446$	$0.9734 \pm 0.0525$	$0.9696 \pm 0.0369$	$0.9601 \pm 0.0322$	$0.8843 \pm 0.0919$
	Sen	$\underline{0} .\underline{9571} \pm \underline{0} .\underline{0964}$	$0.9232 \pm 0.0887$	$0.9304 \pm 0.0736$	$0.9446 \pm 0.0983$	$0.8786 \pm 0.0436$	$0.7411 \pm 0.1399$
	Prec	$\underline{0} .\underline{9589} \pm \underline{0} .\underline{0945}$	$0.8913 \pm 0.1026$	$0.9069 \pm 0.1189$	$0.8783 \pm 0.1274$	$0.8682 \pm 0.1247$	$0.7994 \pm 0.1370$
Fusion #3	ACC%	$94.49\% \pm 7.47\%$	$85.48 \% \pm 9.86\%$	-	-	-	-
	AUC	$0.9860 \pm 0.0353$	$0.9523 \pm 0.0570$	-	-	-	-
	Sen	$0.9304 \pm 0.0998$	$0.8161 \pm 0.1473$	-	-	-	-
	Prec	$0.9478 \pm 0.0957$	$0.8718 \pm 0.1454$	-	-	-	-

The results shown in italic are of experiments that are not explored in the original research papers

The underlined classification results represent the highest results for each corresponding section

## Abbreviations

RP: Relative phase
SVM: Support vector machine
DTB: Decision tree bagger
ANOVA: Analysis of variance
CNN: Convolutional neural network
DL: Deep learning
CM: Co-occurrence matrix
LBP: Local binary patterns
LOSIB: Local oriented statistics information booster
SFTA: Segmentation-based fractal texture analysis
BreakHis: Breast cancer histopathological dataset
SH: Shearlet
PD: Pixel distance
PCA: Principal component analysis
QP: Quadratic programming
CV: Cross-validation
Perf: Performance
ACC: Accuracy
AUC: Area under the receiver operating characteristic curve
Sen: Sensitivity
Prec: Precision

## References

[1] Irshad H, Veillard A, Roux L, Racoceanu D (2014). Methods for nuclei detection, segmentation, and classification in digital histopathology: a review–current status and future potential. IEEE Rev Biomed Eng.

[2] Xu J, Xiang L, Liu Q, Gilmore H, Wu J, Tang J, Madabhushi A (2016). Stacked sparse autoencoder (SSAE) for nuclei detection on breast cancer histopathology images. IEEE Trans Med Imaging.

[3] Spanhol FA, Oliveira LS, Petitjean C, Heutte L (2016). A dataset for breast cancer histopathological image classification. IEEE Trans Biomed Eng.

[4] Kather JN, Weis C-A, Bianconi F, Melchers SM, Schad LR, Gaiser T, Marx A, Zöllner FG (2016). Multi-class texture analysis in colorectal cancer histology. Sci Reports.

[5] Linder N, Konsti J, Turkki R, Rahtu E, Lundin M, Nordling S, Haglund C, Ahonen T, Pietikäinen M, Lundin J (2012). Identification of tumor epithelium and stroma in tissue microarrays using texture analysis. Diagn Pathol.

[6] Bruno DOT, do Nascimento MZ, Ramos RP, Batista VR, Neves LA, Martins AS, Lbp operators on curvelet coefficients as an algorithm to describe texture in breast cancer tissues. Expert Syst Appl. 2016;55:329–40.

[7] Ribeiro MG, Neves LA, do Nascimento MZ, Roberto GF, Martins AS, Tosta TAA (2019). Classification of colorectal cancer based on the association of multidimensional and multiresolution features. Expert Syst Appl.

[8] Vo AP, Oraintara S, Nguyen TT. Using phase and magnitude information of the complex directional filter bank for texture image retrieval. In: 2007 IEEE International Conference on Image Processing, 2007;4, p. 61. IEEE.

[9] He J, Ji H, Yang X (2013). Rotation invariant texture descriptor using local shearlet-based energy histograms. IEEE Signal Process Lett.

[10] Zhou S, Shi J, Zhu J, Cai Y, Wang R (2013). Shearlet-based texture feature extraction for classification of breast tumor in ultrasound image. Biomed Signal Process Control.

[11] Dong Y, Tao D, Li X, Ma J, Pu J (2015). Texture classification and retrieval using shearlets and linear regression. IEEE Trans Cybernet.

[12] Meshkini K, Ghassemian H. Texture classification using Shearlet transform and GLCM. In: 2017 Iranian Conference on Electrical Engineering (ICEE), 2017;1845–1850. IEEE.

[13] Song Y, Chang, H, Gao Y, Liu S, Zhang D, Yao J, Chrzanowski W, Cai W. Feature learning with component selective encoding for histopathology image classification. In: IEEE 15th International Symposium on Biomedical Imaging (ISBI 2018), 2018;257–260. IEEE.

[14] Gupta V, Bhavsar A. Sequential modeling of deep features for breast cancer histopathological image classification. In: Proceedings of the IEEE Conference on Computer Vision and Pattern Recognition Workshops, 2018;2254–2261.

[15] Wang C, Shi J, Zhang Q, Ying S. Histopathological image classification with bilinear convolutional neural networks. In: 2017 39th Annual International Conference of the IEEE Engineering in Medicine and Biology Society (EMBC), 2017;4050–4053. IEEE. 10.1109/EMBC.2017.8037745 29060786

[16] Alinsaif S, Lang J. Histological image classification using deep features and transfer learning. In: 2020 17th Conference on Computer and Robot Vision (CRV), 2020;101–108. IEEE.

[17] Qu J, Hiruta N, Terai K, Nosato H, Murakawa M, Sakanashi H. Gastric pathology image classification using stepwise fine-tuning for deep neural networks. J Healthc Eng. 2018;2018:10.1155/2018/8961781 PMC6033298 30034677

[18] Therrien R, Doyle S. Role of training data variability on classifier performance and generalizability. In: Medical Imaging 2018: Digital Pathology, vol. 10581, p. 1058109 (2018). International Society for Optics and Photonics.

[19] Soh L-K, Tsatsoulis C (1999). Texture analysis of SAR sea ice imagery using gray level co-occurrence matrices. IEEE Trans Geosci Remote Sens.

[20] Haralick RM, Shanmugam K (1973). Others: textural features for image classification. IEEE Trans Syst Man Cybern.

[21] Ojala T, Pietikäinen M, Mäenpää T (2002). Multiresolution gray-scale and rotation invariant texture classification with local binary patterns. IEEE Trans Pattern Anal Mach Intell.

[22] García-Olalla O, Alegre E, Fernández-Robles L, González-Castro V. Local oriented statistics information booster (losib) for texture classification. In: 2014 22nd International Conference on Pattern Recognition, 2014;1114–1119. IEEE.

[23] Costa AF, Humpire-Mamani G, Traina AJM. An efficient algorithm for fractal analysis of textures. In: 2012 25th SIBGRAPI Conference on Graphics, Patterns and Images (SIBGRAPI), pp. 39–46 (2012). IEEE.

[24] Kutyniok G, Labate D. Introduction to shearlets. Shearlets. 2012;1–38:

[25] Cristianini N, Shawe-Taylor J (2000). An introduction to support vector machines and other kernel-based learning methods.

[26] Breiman L (1996). Bagging predictors. Mach Learn.

[27] Breiman L, Friedman J, Stone CJ, Olshen RA (1984). Classification and regression trees.

[28] Došilović FK, Brčić M, Hlupić N. Explainable artificial intelligence: a survey. In: 2018 41st International Convention on Information and Communication Technology, Electronics and Microelectronics (MIPRO), 2018;0210–0215. IEEE.

[29] Alinsaif S, Lang J. Shearlet-based techniques for histological image classification. In: 2019 IEEE International Conference on Bioinformatics and Biomedicine (BIBM); 2019.

[30] Jonnalagedda P, Schmolze D, Bhanu B. [regular paper] mvpnets: Multi-viewing path deep learning neural networks for magnification invariant diagnosis in breast cancer. In: 2018 IEEE 18th International Conference on Bioinformatics and Bioengineering (BIBE), 2018;189–194. IEEE.

[31] Sellaro TL, Filkins R, Hoffman C, Fine JL, Ho J, Parwani AV, Pantanowitz L, Montalto M (2013). Relationship between magnification and resolution in digital pathology systems. J Pathol Inf.

[32] Sirinukunwattana K, Pluim JP, Chen H, Qi X, Heng P-A, Guo YB, Wang LY, Matuszewski BJ, Bruni E, Sanchez U (2017). Gland segmentation in colon histology images: the glas challenge contest. Med Image Anal.

[33] Reisenhofer R. The complex shearlet transform and applications to image quality assessment. Master’s thesis, Technische Universität Berlin 2014.

[34] Candès EJ, Donoho DL (2004). New tight frames of curvelets and optimal representations of objects with piecewise c2 singularities. Commun Pure Appl Math J Issued Courant Inst Math Sci.

[35] Do MN, Vetterli M (2005). The contourlet transform: an efficient directional multiresolution image representation. IEEE Trans Image Process.

[36] Kutyniok G, Shahram M, Zhuang X (2012). Shearlab: a rational design of a digital parabolic scaling algorithm. SIAM J Imaging Sci.

[37] Clausi DA (2002). An analysis of co-occurrence texture statistics as a function of grey level quantization. Can J Remote Sens.

[38] Pan Y, Liu L, Yang L, Wang Y. Texture feature extracting method based on local relative phase binary pattern. In: 2016 5th International Conference on Computer Science and Network Technology (ICCSNT), 2016;749–753. IEEE.

[39] Cai L, Wang X, Wang Y, Guo Y, Yu J, Wang Y (2015). Robust phase-based texture descriptor for classification of breast ultrasound images. Biomed Eng Online.

[40] Oppenheim AV, Lim JS (1981). The importance of phase in signals. Proc IEEE.

[41] Harman HH (1976). Modern factor analysis.

[42] Stegmann MB, Sjöstrand K, Larsen R. Sparse modeling of landmark and texture variability using the orthomax criterion. In: Medical Imaging 2006: Image Processing, 2006;6144: 61441. International Society for Optics and Photonics.

[43] Zanaty E (2012). Support vector machines (svms) versus multilayer perception (mlp) in data classification. Egyptian Inf J.

[44] Hastie T, Tibshirani R. Classification by pairwise coupling. In: Advances in Neural Information Processing Systems, 1998;507–513.

[45] Platt J. Sequential minimal optimization: a fast algorithm for training support vector machines; 1998.

[46] Friedman J, Hastie T, Tibshirani R (2001). The elements of statistical learning.

[47] Breiman L (2001). Random forests. Mach Learn.

[48] Ramalho GLB, Ferreira DS, Rebouças Filho PP, de Medeiros FNS (2016). Rotation-invariant feature extraction using a structural co-occurrence matrix. Measurement.

[49] Gunning D. Explainable artificial intelligence (xai). Defense Advanced Research Projects Agency (DARPA), nd Web 2017;2.

